# The VarA-CsrA regulatory pathway influences cell shape in *Vibrio cholerae*

**DOI:** 10.1371/journal.pgen.1010143

**Published:** 2022-03-28

**Authors:** Leonardo F. Lemos Rocha, Katharina Peters, Jacob Biboy, Jamie S. Depelteau, Ariane Briegel, Waldemar Vollmer, Melanie Blokesch

**Affiliations:** 1 Laboratory of Molecular Microbiology, Global Health Institute, School of Life Sciences, École Polytechnique Fédérale de Lausanne (EPFL), Lausanne, Switzerland; 2 Centre for Bacterial Cell Biology, Biosciences Institute, Newcastle University, Newcastle Upon Tyne, United Kingdom; 3 Microbial Sciences, Institute of Biology, Leiden University, Leiden, The Netherlands; Universidad de Sevilla, SPAIN

## Abstract

Despite extensive studies on the curve-shaped bacterium *Vibrio cholerae*, the causative agent of the diarrheal disease cholera, its virulence-associated regulatory two-component signal transduction system VarS/VarA is not well understood. This pathway, which mainly signals through the downstream protein CsrA, is highly conserved among gamma-proteobacteria, indicating there is likely a broader function of this system beyond virulence regulation. In this study, we investigated the VarA-CsrA signaling pathway and discovered a previously unrecognized link to the shape of the bacterium. We observed that *varA*-deficient *V*. *cholerae* cells showed an abnormal spherical morphology during late-stage growth. Through peptidoglycan (PG) composition analyses, we discovered that these mutant bacteria contained an increased content of disaccharide dipeptides and reduced peptide crosslinks, consistent with the atypical cellular shape. The spherical shape correlated with the CsrA-dependent overproduction of aspartate ammonia lyase (AspA) in *varA* mutant cells, which likely depleted the cellular aspartate pool; therefore, the synthesis of the PG precursor amino acid meso-diaminopimelic acid was impaired. Importantly, this phenotype, and the overall cell rounding, could be prevented by means of cell wall recycling. Collectively, our data provide new insights into how *V*. *cholerae* use the VarA-CsrA signaling system to adjust its morphology upon unidentified external cues in its environment.

## Author summary

Responsible for the diarrheal disease cholera, the bacterium *Vibrio cholerae* tightly regulates its virulence program according to external stimuli. Here, we discovered that a sensing-response mechanism involved in the regulation of virulence also controls bacterial shape. We show that *V*. *cholerae* lacking this system lose their normal comma shape and become spherical due to an abnormal cell wall composition caused by metabolic changes that reduce available cell wall building blocks. Our study therefore sheds new light on how *V*. *cholerae* potentially modulates its morphology based on environmental changes.

## Introduction

The current ongoing 7^th^ cholera pandemic sickens millions of people every year [1], though many questions still surround the pathogenicity of its causative agent, the well-studied gram-negative bacterium *Vibrio cholerae*. *V*. *cholerae* is frequently found in aquatic habitats [2], but throughout human history, it has caused several cholera pandemics, leading to questions about how it switched from an environmental to a pathogenic lifestyle. Nonetheless, it is well established that virulence induction is linked to the ability of the bacterium to sense its surroundings [3]. Hence, it is important to study the mechanisms that allow bacteria to detect environmental changes and rapidly adapt to them.

One example of such a sensing mechanism is the **v**irulence-**a**ssociated **r**egulators S and A (VarS/VarA) two-component system (TCS) of *V*. *cholerae*. For this TCS, the sensor kinase VarS detects an unknown signal and activates the response regulator VarA through phosphotransfer. Subsequently, phosphorylated VarA binds to the promoter regions of the genes that encode the small RNAs (sRNAs) *csrB*, *csrC*, and *csrD*, fostering their transcription. These sRNAs control the activity of carbon storage regulator A (CsrA) by sequestering it away from its mRNA targets [4]. This sRNA-based sequestration mechanism is highly conserved among gamma-proteobacteria and known to be important beyond virulence regulation in those organisms [5]. CsrA is a post-transcriptional regulator that binds to consensus motifs of specific mRNAs, thereby controlling access to the ribosome binding site, which ultimately promotes or prevents translation [6]. Additionally, CsrA controls the formation of RNA hairpins, which can expose Rho-binding sites, leading to premature transcriptional termination. Furthermore, CsrA controls mRNA stability by preventing mRNA cleavage by the endonuclease RNase E [6] and it has the flexibility to bind mRNAs in different conformational states, thereby increasing the number of genes that can be modulated by this global regulator [7].

RNA-based regulatory systems often control virulence in pathogenic gamma-proteobacteria [8]. Indeed, for *V*. *cholerae*, the VarS/VarA system was regarded as a virulence-associated regulatory TCS, given that a *varA* mutant produced reduced levels of the two major virulence factors (e.g., the cholera toxin and the toxin co-regulated pilus) compared to its parental wildtype (WT) strain. This reduced production of virulence factors resulted in an *in vivo* fitness disadvantage of the mutant compared to the WT upon intestinal colonization of infant mice [9]. The VarS/VarA system also contributes to the dissemination of *V*. *cholerae* from the host into the environment [10]. Moreover, in conjunction with CsrA, this TCS is known to be involved in the regulation of central carbon metabolism, iron uptake, lipid metabolism, flagellum-dependent motility, and other phenotypes [11,12]. Collectively, signaling through the VarS/VarA-CsrA circuit therefore affects the environmental lifestyle of *V*. *cholerae* as well as its pathogenesis.

The VarS/VarA system is also involved in quorum sensing (QS) [4,13], which is a cell-to-cell communication process mediated by the secretion, accumulation, and sensing of extracellular signaling molecules (autoinducers). This process fosters synchronized bacterial behavior, such as bioluminescence, biofilm production, competence for DNA uptake, and virulence regulation [14]. In *V*. *cholerae*, the master regulator of QS, HapR, is produced at high cell densities. Previous work has shown that VarA controls HapR abundance, as this QS regulator was undetectable in the absence of *varA* [4,13]. However, the exact mechanism behind this regulation is still not fully understood.

Here, we aimed to better understand the VarA-CsrA signaling pathway to explore the role of this sensing system on the emergence of virulence in *V*. *cholerae*. In this work, we identified a new role of this regulatory system in the modulation of the cellular morphology of *V*. *cholerae*. More precisely, we show that its normal morphology is lost in *varA*-deficient strains, resulting in round instead of curve-shaped cells during prolonged growth. This spherical morphology is likely the result of a weakened peptidoglycan (PG) cell wall due to reduced numbers of peptide cross-linkages. Moreover, we demonstrate that the changed PG composition is likely a consequence of a lack of cell wall precursors due to the overproduction of the aspartate ammonia lyase enzyme. Collectively, this study deciphers how the VarA-CsrA pathway regulates cell shape by modulating cell metabolism, and therefore, cell wall building blocks, giving more insight to the bacterial regulation of a pathway required for virulence.

## Results and discussion

### VarA deficiency results in cell rounding

**Fig 1:**
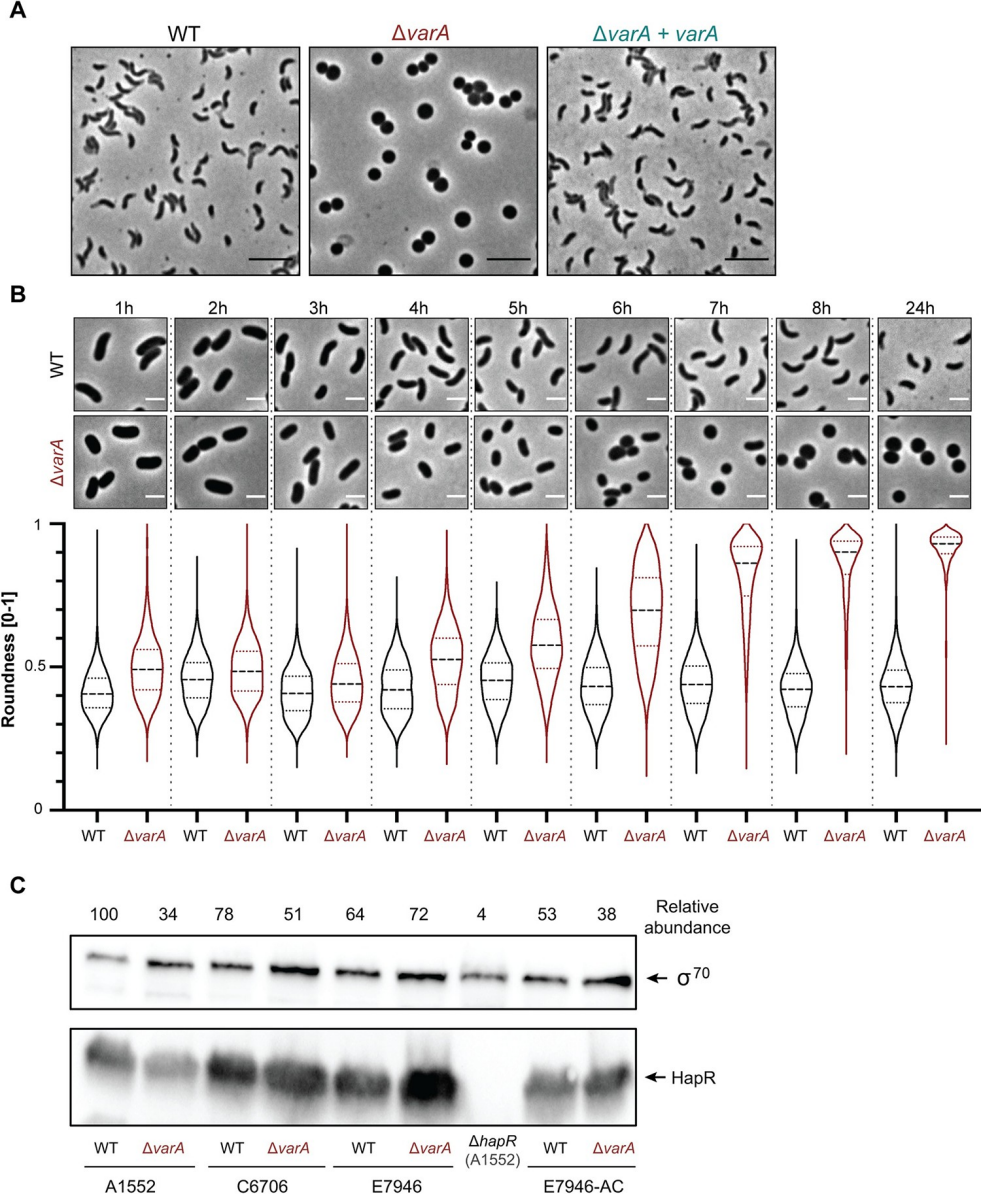
*V*. *cholerae* Δ*varA* cells change their morphology at later stages of growth. (A) Δ*varA* cells grown overnight are round. Phase contrast micrographs of the WT, Δ*varA*, and the complemented Δ*varA*+*varA* strains that were grown for 20 h. Scale bar: 5 μm. (B) Δ*varA* cells become round during late growth. Phase contrast microscopy imaging (top) and quantification (roundness values at the bottom) of the WT and Δ*varA* strains during growth. Cells were imaged every hour for 8 h and again at 24 h post-dilution. Scale bar: 2 μm. The roundness was quantified using the MicrobeJ software and is based on 3,000 cells each (n = 1000 per biologically independent experiment). (C) Δ*varA* cells produce the QS regulatory protein HapR. Western blot analysis to detect the HapR protein levels in the different *V*. *cholerae* El Tor pandemic strains A1552, C6707, E7946, and their *varA-*deficient mutants, with the A1552Δ*hapR* strain as a negative control (strain details are provided in S4 Table). All strains were sampled at an OD_600_ of ~2.5. Representative blot from three independent experiments. Detection of σ^70^ served as a loading control. The densitometric quantification of the HapR abundance is shown above the blots (normalized to the σ^70^ signal and to the WT (A1552), which was set to 100).

Given that VarS/VarA are a conserved regulatory pathway among gamma-proteobacteria [5], we sought to study the contribution of this TCS towards diverse *V*. *cholerae* cellular processes. Unexpectedly, while generating mutants of this TCS, we observed that *varA*-deficient *V*. *cholerae* cells exhibited an unusual cell morphology (Fig 1A). Instead of the comma-like shape common for most *Vibrio* species and that was observed for the parental WT strain, the *varA* mutant cells were found to be round in shape after overnight growth. Complementation of the mutant through the provision of the promoter-preceded *varA* gene *in cis* (Δ*varA*+*varA*) restored the WT morphology (Fig 1A), supporting the causality between VarA deficiency and the changed cell shape.

To investigate the shape of a larger number of bacteria while reducing image selection bias, we implemented MicrobeJ as a tool for quantitative single cell microscopy image analysis [15]. This software can measure the *roundness* of a cell, with a parameter value of 0 representing a straight line and 1 a perfect sphere. The analysis of 3000 WT *V*. *cholerae* cells showed that the majority of the cells depicted a roundness value of ~0.4 (S1 Fig). In contrast, the round-shaped Δ*varA* cells showed values close to 1, confirming a nearly spherical cell shape of the mutant. Upon complementation, the measured parameter decreased towards the values of the WT (S1 Fig). Based on the length and width values obtained by MicrobeJ, we also estimated the cells’ volume. This analysis indicated that the spherical shape of the Δ*varA* bacteria resulted in an increased cell volume compared to the *Vibrio*-shaped WT cells (S2 Fig).

Given that VarA is thought to primarily act downstream of the histidine kinase VarS, we next assessed the morphology of *varS*-deficient cells. Interestingly, this mutant strain did not phenocopy the Δ*varA* morphology (S1 Fig), even though an intermediate phenotype was occasionally observed. Additionally, a Δ*varS*Δ*varA* double mutant was morphologically indistinguishable from the *varA* single mutant (S1 Fig). The difference between the phenotypes of the *varA* and *varS* mutants suggests that VarA can act independently of VarS. To test this assumption, we measured the expression of the downstream genes encoding for the sRNA (e.g., *csrB*, *csrC*, and *csrD*) as a read-out for VarS and VarA activity (S3 Fig). While the absence of *varA* caused a significant reduction of these genes’ transcripts compared to the WT, the Δ*varS* strain exhibited an intermediate phenotype (S3 Fig). This finding is consistent with previous work by Lenz *et al*. who demonstrated a 10-fold stronger regulatory effect of VarA compared to VarS and a partially VarS-independent but VarA-dependent regulation of the sRNA genes *csrB* and *csrD* [4]. Moreover, it is known that the homologous system GacS/GacA in *Pseudomonas aeruginosa* forms a multicomponent signal transduction system with other histidine kinases [16]. As such, additional histidine kinases might also exist in *V*. *cholerae* that bypass VarS.

### VarA-dependent cell morphology is growth-phase specific

For further insight into the underlying dynamics of the morphological phenotype of the Δ*varA* mutant, we examined whether rounding occurred at a particular point during growth by performing time-course experiments. Here, we assessed cellular morphology every hour, including a 24 h control sample. As shown in Fig 1B, the *varA-*deficient cells initially maintained a WT-like morphology, which started to change around 4 h post-dilution. From this point onwards, the roundness of the cells continuously increased until they reached peak roundness at 8 h. We did observe a growth defect for the Δ*varA* strain compared to WT (as shown by the enumeration of colony-forming units [CFU] per ml and OD_600_ measurements; S4 Fig). However, after 24 h of growth, the *varA* mutant reached similar values as the WT (S4 Fig). Given the fact that the WT bacteria maintained their *Vibrio* shape throughout the duration of the experiment, we excluded the growth delay as the (sole) reason for the changed morphology of the *varA-*deficient cells (Fig 1B).

We did not observe any division of the spherical cells. Upon dilution into fresh medium, however, round-shaped Δ*varA* bacteria started to elongate followed by a division into WT-shaped daughter cells, as documented by time-lapse microscopy (S1 Movie). Collectively, we concluded that the abnormal cell shape of the *varA* mutant was growth-phase dependent and most prominent at later time points during growth. These data therefore suggest a previously unknown role of VarA in cell shape maintenance during the stationary phase of *V*. *cholerae*.

### Quorum sensing (QS) is maintained in *varA*-deficient cells

Given the timing of the observed shape transition phenotype and the reported role of VarA in the production of HapR [4,13], we asked whether the cell rounding was linked to a HapR deficiency, and therefore, a QS defect. However, to our surprise and in contrast to previous reports [4,13], the Δ*varA* cells still produced copious amounts of the HapR protein (Fig 1C). We confirmed this finding by deleting *varA* in two additional pandemic O1 El Tor *V*. *cholerae* strains (C6706 and E7946) and by testing a previously published *varA* mutant from the Camilli laboratory [10] (E7946-AC; Fig 1C). In addition to the production of HapR in these strains, we observed that deleting *hapR* in *varA*-deficient *V*. *cholerae* partially reduced the cell rounding phenotype. These results further support the notion that HapR is present and active in Δ*varA* cells (S5A Fig). Notwithstanding, deleting *luxO*, whose gene product indirectly acts as a repressor of HapR synthesis at low cell density, did not change the rounding phenotype of the *varA*-deficient cells (S5B Fig).

To follow up on the inconsistency of the observed HapR production in *varA*-deficient cells compared to previous studies [4,13], we wondered if this could be linked to a frequently used QS-impaired lab domesticated variant of *V*. *cholerae* (strain C6706) [17]. In previous work, we found that such QS-impaired domesticated variants differ from wild bacteria, and that much of the research done on these strains does not accurately reflect the behavior of QS-proficient strains [17]. Indeed, in the case of the C6706 variant, a gain-of-function (GOF) mutation in *luxO* (encoding LuxO[G333S]) lowers *hapR* transcript levels even at a high cell density, which is known to mask many important phenotypes [17]. We therefore introduced the *luxO* GOF mutation into strains A1552 and scored HapR levels in the presence or absence of *varA* (S5C Fig). For comparison, we also included the original C6706 strain (before it was rendered streptomycin resistant; kind gift from J.J. Mekalanos) and a representative sample of its lab-domesticated variant (named here C6706-mut) and their Δ*varA* mutants in this analysis (S5C Fig). These data showed that the already low HapR levels in those strains that carried the *luxO*[G333S] GOF mutation were indeed diminished beyond the level of detection upon deletion of *varA*, which likely explains the discrepancy between our results and previous reports [4,13]. However, in the presence of native, non-mutated *luxO*, deleting *varA* did not abrogate HapR production. We therefore conclude that a defective QS circuit is unlikely to be responsible for the Δ*varA* cell morphology.

### VarA deficiency does not phenocopy the VBNC state

Since the abnormal morphology of *varA*-deficient cells occurred at the stationary phase, we wondered whether cells were entering the viable but non-culturable (VBNC) state. This state is usually triggered by restrained metabolic activity caused by, for instance, low nutrients availability and/or an extended time at low temperatures [18]. Cells that enter this state are round in shape due to multiple morphological changes, such as dehiscence of the inner and outer membranes and the resulting enlargement of the periplasmic space [19]. To test whether *ΔvarA* cells had similar morphological defects, we imaged *varA*-deficient cells and their parental WT cells after 20 h of growth by cryo-electron microscopy. As shown in S6 Fig, neither the size of the periplasm nor the morphology of the membranes appeared significantly altered in the *varA* mutant compared to the WT. Collectively, and taken together with the fact that the bacteria maintained their culturability, these observations suggest that the rounding phenotype of Δ*varA* cells differed from the well-described *V*. *cholerae* VBNC state.

### Spherical-shaped *varA*-deficient cells have an abnormal PG

**Fig 2:**
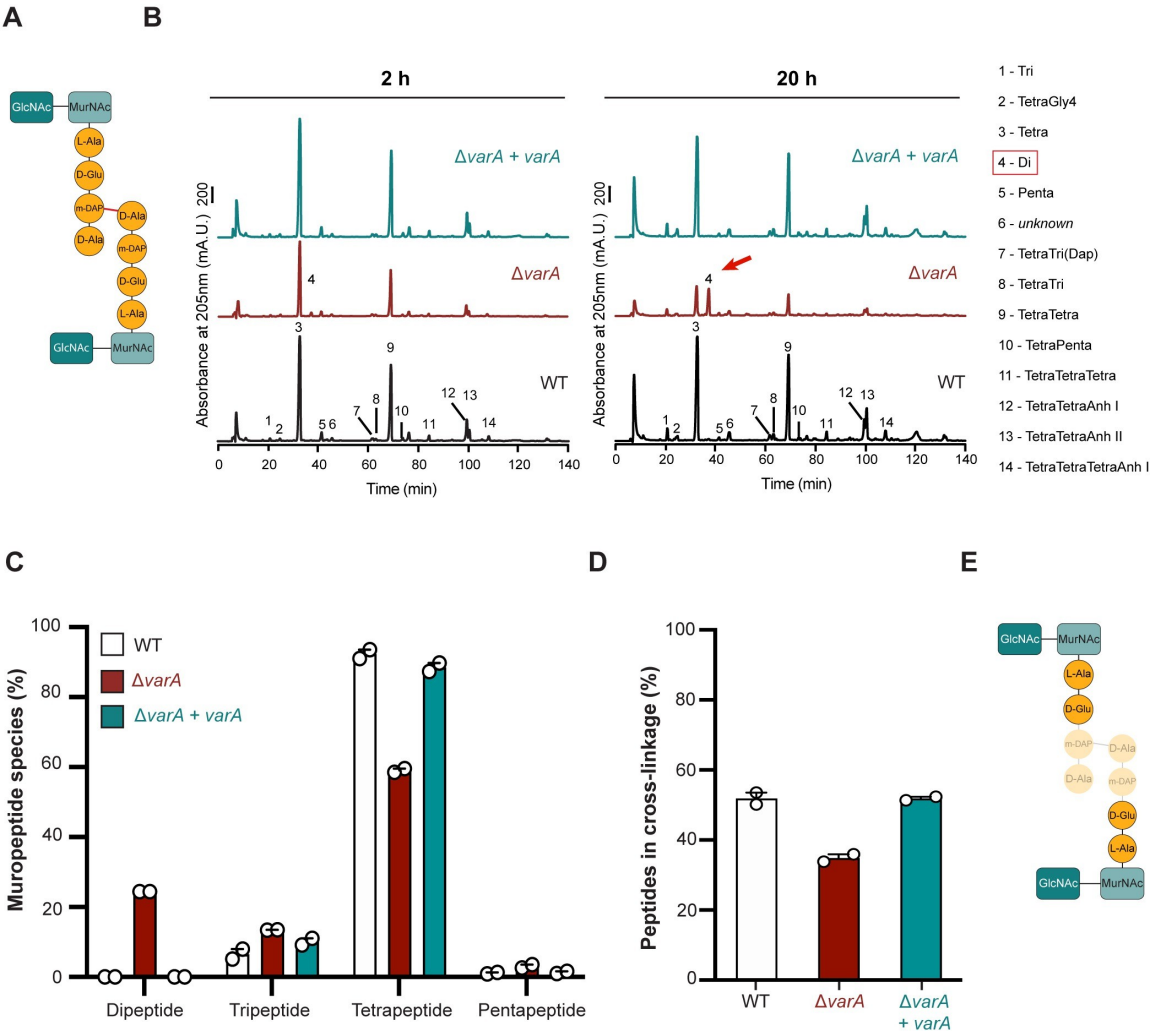
The Δ*varA* mutant has an unusual PG composition. (A) Scheme of the peptide cross-link in PG. GlcNAc–N-acetylglucosamine; MurNAc–N-acetylmuramic acid; L-Ala–L-alanine; D-Glu–D-glutamic acid; m-DAP–meso-diaminopimelic acid; D-Ala–D-alanine. (B) The Δ*varA* strain contains a new muropeptide and less peptide cross-links. High performance liquid chromatograms of purified PG from the WT, Δ*varA*, and complemented Δ*varA*+*varA* strains isolated after 2 h or 20 h of growth, followed by digest with cellosyl. Numbered peaks correspond to each muropeptide indicated on the right. (C) The Δ*varA* strain contains dipeptides in the PG. Quantification of the different peptides in the purified PG of the WT, Δ*varA*, and complemented Δ*varA*+*varA* strains isolated at 20 h post-dilution. (D) The Δ*varA* strain has less peptide cross-links. Percentage of cross-linked peptides in purified PG derived from the WT, Δ*varA*, and Δ*varA*+*varA* strains after 20 h of growth. For (C) and (D): Values are mean (± variance of two biological replicates). See S1 Table for details. (E) Scheme illustrating that dipeptides replace peptide cross-links in the PG of the Δ*varA* strain.

Given the absence of any obvious morphological defects of the inner and outer membranes and the periplasm, we speculated that the absence of VarA might impair the PG fine-structure, as this mesh-like polymer is known to be required to maintain cell shape [20]. The PG is located between the inner and outer membranes in gram-negative bacteria and composed of glycan chains of alternating N-acetylglucosamine (GlcNAc) and N-acetylmuramic acid (MurNAc) residues (Fig 2A). The poly-GlcNAc-MurNAc glycan chains are connected by peptides and both together form the sacculus around the inner membrane [20]. Hence, we isolated the PG from the WT, Δ*varA*, and complemented Δ*varA*+*varA* strains, digested it with the muramidase cellosyl and analyzed the composition of the muropeptides by High Performance Liquid Chromatography (HPLC). We collected data at two different time-points: (1) at 2 h post-dilution, when the cell shape of the *varA* mutant resembles that of the WT; and (2) at 20 h post-dilution, when the mutant exhibits its round morphology. Interestingly, the analysis of the isolated PG showed an unusual composition in *varA*-deficient cells at 20 h post-dilution (Fig 2B–2D and S1 Table). Specifically, a new peak (number 4; red arrow in Fig 2B) appeared exclusively in the chromatogram of the Δ*varA* samples and was strongly increased in the 20 h sample (to 24.4±0.0% of total muropeptides), while this peak was below detection limit in the samples from the wild-type or complemented mutant strain (Fig 2C and S1 Table). Mass spectrometry analysis confirmed this peak as disaccharide-L-Ala-D-Glu (Di) (Fig 2B and S1 Table). The high abundance of these dipeptides (24.4±0.0%) came at the expense of the tetrapeptides, which were significantly reduced in the Δ*varA* cells (Fig 2C and S1 Table; from 92.4±5.9% abundance in late-grown WT down to 59.1±3.9% in the late-growth Δ*varA* mutant). Importantly, the PG of the 20 h Δ*varA* sample had significantly reduced peptide cross-linkage with 34.9±1.1% peptides present in cross-links compared to 51.8±1.7% and 51.9±0.5% in the wild-type and *varA-*complemented strain, respectively (Fig 2D and S1 Table). Hence, we conclude that the round shape of the Δ*varA* strain at the late growth stages is the result of a weakened PG caused by the high abundance of dipeptides and reduced peptide cross-linkage (Fig 2E).

To our knowledge, such a high abundance of dipeptides was never observed in *V*. *cholerae*. One hypothesis to explain this phenotype could be the misregulation of an endopeptidase that cleaves between the second and third amino acids in the peptides. It is known, for instance, that a DL-carboxypeptidase (Pgp1) cleaves disaccharide tripeptides into dipeptides in *Campylobacter jejuni* [21]. In *C*. *jejuni*, the deletion or overexpression of Pgp1 prevents the normal helical morphology of the bacterium as a result of the higher and lower abundance of dipeptides, respectively [21]. More recently, Pgp1 was also shown to be involved in the *C*. *jejuni* helical-coccoid transition after extended incubation or starvation [22]. We therefore inspected the annotated genome of *V*. *cholerae*, but could not identify any obvious DL-carboxypeptidase gene. Interestingly, the amidase AmiA of *Helicobacter pylori* was also shown to foster an accumulation of dipeptides, which correlated with the bacterial transition from a bacillary form to a coccoid morphology [23]. Likewise, Möll *et al*. showed that the absence of the amidase AmiB, which is usually responsible for the cleavage of septal PG, led to an increase of dipeptides (~7% compared to < 1% for the WT) in *V*. *cholerae* [24]. However, this deletion of *amiB* in *V*. *cholerae* resulted in filamentous cells due to their inability to divide properly [24], which is significantly distinguished from the Δ*varA* phenotype described above. We therefore speculate that the altered PG in this mutant is not caused by a misregulated enzyme.

### WT-derived PG building blocks prevent rounding of Δ*varA* cells

**Fig 3:**
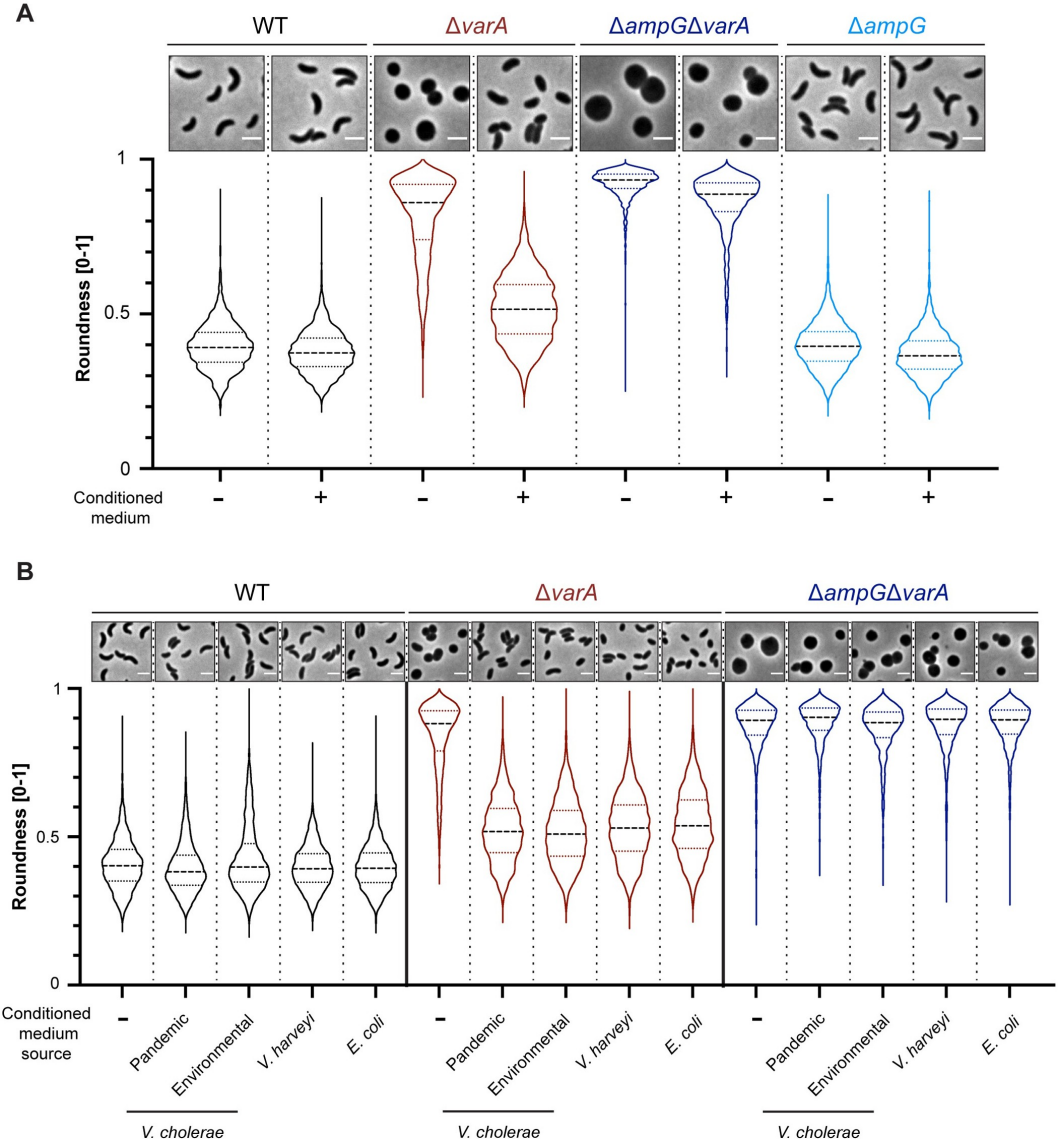
Recycling of PG prevents rounding of Δ*varA* cells. (A) Conditioned medium rescues the *Vibrio* shape of Δ*varA* in an AmpG-dependent manner. Phase contrast micrographs (top) and shape quantification (bottom) of the WT, Δ*varA*, Δ*ampG*Δ*varA*, and Δ*ampG* strains grown in the absence (-) or presence (+) of conditioned medium. Scale bar: 2 μm. The quantification of roundness was performed with 3000 cells (n = 1000 per biologically independent experiment) using the MicrobeJ software. Roundness values range from 0 to 1. (B) Conditioned medium of diverse gram-negative bacteria rescues the Δ*varA* rounding phenotype. Conditioned medium was derived from pandemic *V*. *cholerae* A1552 or environmental *V*. *cholerae* SA5Y, *V*. *harveyi*, or *E*. *coli*. Details as in panel A.

As we were unable to identify any obvious peptide-cleaving enzyme, we instead hypothesized that the altered PG composition of the Δ*varA* cells could be caused by a lack of PG precursors. Irnov *et al*. demonstrated that changes in cell metabolism caused by a deletion of *hfq* in *Caulobacter crescentus* impaired the synthesis of meso-diaminopimelic acid (m-DAP). This in turn was shown to cause cell shape defects [25]. Given that bacteria release PG building blocks into their surroundings during cell wall synthesis [26], we wondered whether the cell wall defect of the Δ*varA* strain could be abrogated by WT-derived PG subunits. We therefore grew *varA*-deficient cells in WT-derived conditioned medium, which indeed prevented the rounding of the cells (Fig 3A). To show that this phenotypic rescue was actually dependent on the recycling of the PG subunit, we removed the AmpG permease responsible for the import of muropeptides by generating a *varA/ampG*-deficient double mutant [27]. As predicted, this double mutant could not be rescued by WT-derived conditioned medium (Fig 3A). In contrast, a single *ampG*-deficient mutant showed no cell shape alterations compared to the WT (Fig 3A).

To study whether this phenotype was specific to the pandemic clade of *V*. *cholerae*, we used conditioned media derived from other bacteria, such as an environmental isolate of *V*. *cholerae* (strain SA5Y; [28,29]), *Vibrio harveyi* and *Escherichia coli*. As shown in Fig 3B, conditioned media from any of these bacteria likewise prevented rounding of the Δ*varA* cells. Collectively, these data support the notion that the shape transition of the Δ*varA* strain is caused by a lack of cell wall precursors and that this phenotype can be rescued by PG recycling.

### Defects in *csrA* restore a normal cell shape for the Δ*varA* strain

**Fig 4:**
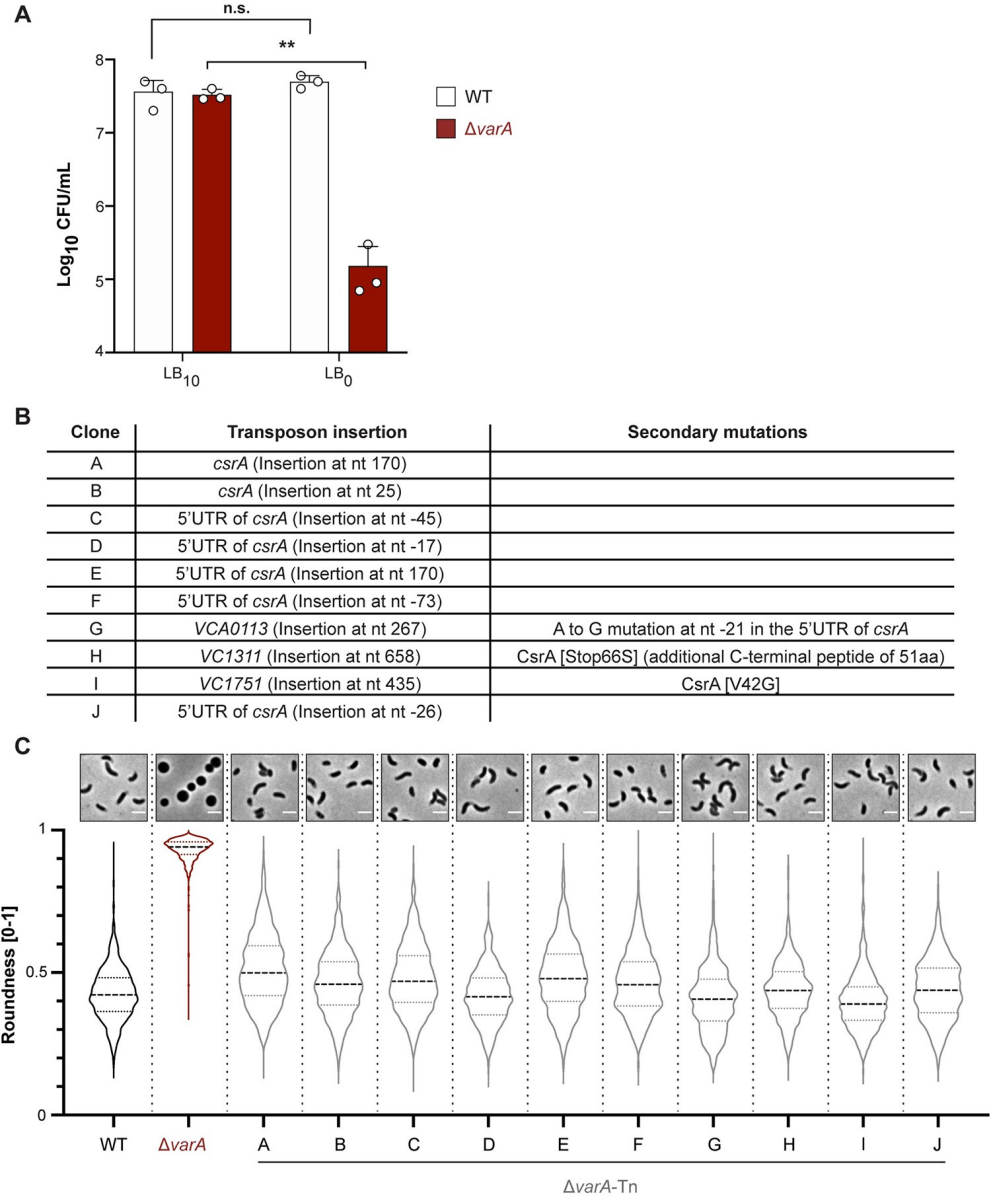
Suppressor mutants of Δ*varA* are linked to *csrA*. (A) Late growth Δ*varA* cells are sensitive to low osmolarity medium. After 20 h of growth, WT and Δ*varA* bacteria were diluted 1:100 in regular LB_10_ or salt-free LB_0_ medium for 1 h before further dilution and plating to enumerate CFUs on the next day. Bars represent the mean of three independent repeats; error bars show the standard deviation (S.D.) Log-transformed data were used for statistical analysis, which were based on unpaired *t* tests with Welch’s correction. **, *P* < 0.01; n.s., not significant. (B) Summary of transposon hits. LB_0_-surviving mutants were isolated from the Δ*varA*-Tn transposon library, and the transposon insertion sites were determined. Information for 10 different mutants is provided. (C) Suppressor mutants restored the *Vibrio* cell shape. Phase contrast micrographs (top) and roundness quantification (bottom) of the WT, Δ*varA*, and the ten Δ*varA*-Tn suppressor mutants (A-J). Cells were imaged at 20 h post-dilution. Imaging details as in Fig 1. Scale bar: 2 μm.

Based on the data provided above, we concluded that the cell rounding of *varA*-deficient cells was caused by a lack of cell wall precursors, which, ultimately, led to a dipeptide-enriched PG mesh with reduced cross-links. However, no link between VarA regulation and the synthesis of cell wall precursors had previously been described for *V*. *cholerae*. To better understand this connection and the underlying signaling pathway, we set up a transposon mutagenesis screen based on the assumption that the weakened cell wall of stationary phase Δ*varA* cells would lyse under osmotic stress. Hence, we generated osmotic stress conditions by incubating the cells in NaCl-free LB medium (LB_0_), as previously reported [30]. This significantly impacted the survival of the mutant cells, while WT cells remained unaffected (Fig 4A).

To next identify suppressor mutants that would survive the LB_0_ treatment, we generated a mariner-based transposon library in the Δ*varA* strain background; these mutants would have a normal PG, and therefore, a comma-shaped morphology. This library was subjected to three consecutive rounds of selection in LB_0_ medium to enrich surviving mutants. Following this experiment, we isolated 24 colonies and identified mutants containing 10 different transposon insertion sites (Δ*varA*-Tn), as shown in Fig 4B. Interestingly, 7 out of 10 mutants had the transposon inserted in or close to *csrA*. To check for any defects in *csrA* in the residual three mutants, we sequenced the *csrA* locus and its flanking regions. We discovered single nucleotide polymorphisms (SNPs) in all of them (Fig 4B). As VarA inhibits CsrA function [4], we therefore concluded that the Δ*varA* morphology was caused by increased CsrA activity due to a lack of VarA~P-dependent expression of the CsrA-sequestering sRNAs *csrB-D*. This finding is consistent with previous work showing that mutations in *csrA* suppressed other Δ*varA* or Δ*varS* phenotypes, including decreased glycogen storage in *V*. *cholerae* or growth defects in *V*. *cholerae* or a Δ*gacA* mutant of *Vibrio fischeri* [10,11,31].

To confirm the selective advantage that these ten *csrA* suppressor mutants might have encountered during the exposure to osmotic stress, we assessed their cell morphology after overnight growth (Fig 4C). Remarkably, all mutants exhibited a (close to) normal comma-like *Vibrio* morphology (Fig 4C), which was based on a restored cell wall composition and crosslinking pattern, as demonstrated for the Δ*varA*-Tn suppressor clone A (S2 Table). Unfortunately, complementation of these suppressor mutants with *csrA* was technically impossible, consistent with previous work that reported a toxic effect of *csrA* expression *in trans* in the absence of functional VarA [11]. Collectively, these findings support the notion that the PG-dependent morphological changes are caused by increased CsrA activity in *varA*-deficient strains.

### Increased CsrA activity causes AspA overproduction

**Fig 5:**
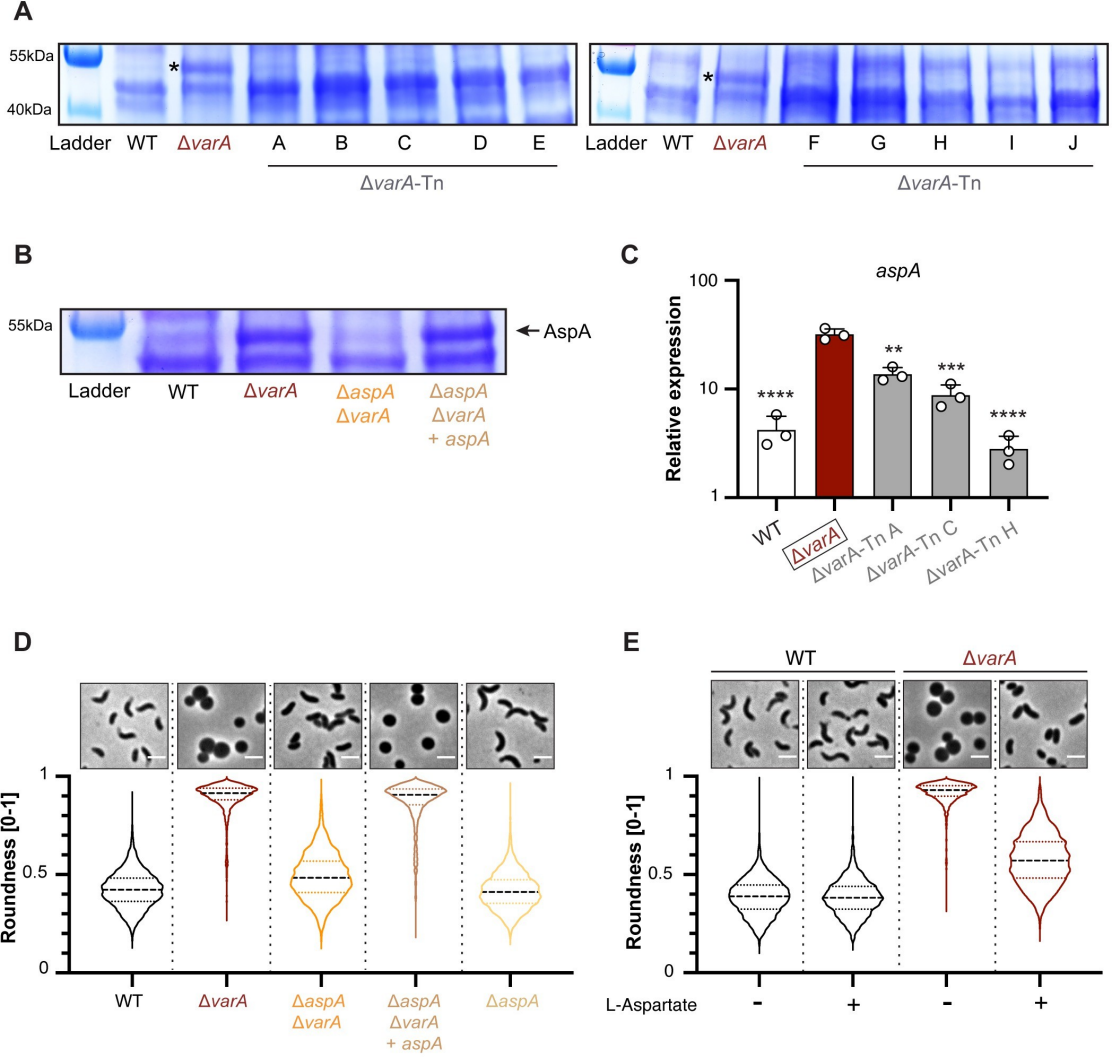
CsrA causes AspA overproduction and impairs m-DAP biosynthesis. (A) The protein pattern is different in the Δ*varA* mutant. Coomassie blue staining of SDS-PAGE-separated proteins from cell lysates of the WT, Δ*varA*, and the Δ*var*A-Tn suppressor mutants A-J. All strains were sampled at an OD_600_ of ~2.5. Bands of the protein ladder are shown in the first lane of each gel and their size is indicated on the left. (B) The dominant protein band corresponds to AspA. Coomassie blue staining of the total cell lysates of the WT, Δ*varA*, Δ*aspA*Δ*varA* and the complemented strain Δ*aspA*Δ*varA*+*aspA*. Details as in panel A. The asterisks (in A) and the arrow (in B) highlight the AspA protein band. (C) CsrA impacts *aspA* expression. Relative expression values for *aspA* transcripts measured in the WT, Δ*varA*, and selected Δ*varA*-Tn suppressor mutants (A, C, and H). All strains were grown to an OD_600_ of ~2.5. The mean values are derived from three independent experiments (shown by individual circles) and the error bars represent the standard deviation. Statistical analyses were performed on log-transformed data and are based on a one-way analysis of variance (ANOVA) test with Dunnett’s multiple comparisons tests. Statistical significance is shown for each sample in comparison to the Δ*varA* strain (boxed). **, *P* < 0.05; ***, *P* < 0.001; ****, *P* < 0.0001. (D and E) Deletion of *aspA* or L-Asp supplementation abrogates rounding in Δ*varA* cells. Phase contrast micrographs (top) and cell shape quantification (bottom) of the WT, Δ*varA*, Δ*aspA*Δ*varA*, and Δ*aspA*Δ*varA*+*aspA* strains (D) or WT and Δ*varA* cells grown in the absence (-) or presence (+) of L-aspartate (L-Asp) (E). Cells were imaged at 20 h post-dilution. Imaging details as in Fig 1 (n = 3000 cells for each condition). Scale bar: 2 μm.

As CsrA is a post-transcriptional regulator [6], we wondered about potential changes at the protein level. We first looked at the global protein composition pattern using whole cell lysates of the WT and Δ*varA* strains. As shown in Fig 5A, a protein band with a size of around 50 kDa became starkly apparent for the *varA*-deficient sample after separation by SDS-PAGE followed by Coomassie blue staining. Importantly, this band was diminished in the ten *csrA* suppressor mutants described above, supporting the role of CsrA in the overproduction of this protein (Fig 5A).

Next, we analyzed this overproduced protein band by mass spectrometry and identified it as aspartate ammonia lyase (AspA). Therefore, we deleted *aspA* in the Δ*varA* background, resulting in the reversal of protein overproduction; additionally, this phenotype was able to be complemented by a new copy of *aspA* (Δ*aspA*Δ*varA*+*aspA*; Fig 5B). Interestingly, previous studies identified the *aspA* mRNA amongst hundreds of direct CsrA targets in both *Salmonella* and *E*. *coli* using the CLIP-seq technique to identify protein-RNA interactions [32,33]. These studies also confirmed CsrA’s role as an important carbon metabolism regulator. For instance, Potts *et al*. provided evidence that CsrA’s binding stabilized the phosphofructokinase (*pfk*) mRNA (in addition to impacting its translation and RNA abundance) thereby activating glycolysis [33]. In our study, we did not test the direct binding of *V*. *cholerae*’s CsrA protein to the *asp* mRNA. However, upon RNA sequencing (RNAseq), we observed that the *aspA* transcript was significantly higher in Δ*varA* compared to the WT cells at an OD_600_ of 2.5 (~24-fold; S3 Table), which we confirmed by quantitative reverse transcription PCR (qRT-PCR; Fig 5C). Notably, the *aspA* mRNA levels were significantly reduced in the Δ*varA* transposon suppressor mutants compared to their Δ*varA* parental strain (Fig 5C). Interestingly, the *aspA* transcript levels were different in each of the three representative mutants (Fig 5C), which most likely reflects residual CsrA functionalities consistent with previous work in which Mey *et al*. described diverse phenotypic responses for a variety of *csrA* suppressor mutations [11]. We therefore conclude that CsrA increases *aspA* transcript levels and, accordingly, AspA production in *V*. *cholerae* even though we didn’t show direct biding of CsrA to the *aspA* mRNA, as reported for *Salmonella* and *E*. *coli* [32,33]. An indirect regulation involving intermediate regulator(s) is therefore formally possible.

### AspA overproduction impairs the synthesis of m-DAP

To verify whether the overproduction of AspA was involved in the changed PG composition, therefore generating the round cell morphology of the Δ*varA* mutant, we imaged the Δ*aspA*Δ*varA* strain after 20 h of growth. As shown in Fig 5D, the *Vibrio* shape was restored in this double mutant, as was the mutants’ PG composition and peptide cross-linkage profile (48.4% for Δ*aspA*Δ*varA* compared to 49.5% for the WT control in this experiment; S2 Table). The *aspA* complemented strain (Δ*aspA*Δ*varA*+*aspA*) retained the spherical morphology characteristic of the Δ*varA* mutant (Fig 5D). Deletion of *aspA* alone did not change the cellular morphology (Fig 5D). Ergo, we conclude that AspA overproduction is involved in the PG-dependent rounding phenotype.

Given that *aspA* deletion restored the WT morphology in *varA*-deficient cells, we wondered why *aspA* mutants were not recovered from our transposon mutagenesis screen (Fig 4B). We hypothesized that putative Δ*varA aspA*-Tn mutants might have retained the Δ*varA* growth defect and would thereby be outcompeted by the *csrA* suppressor mutants. To test this assumption, we monitored the growth of the Δ*varA*Δ*aspA* mutant over 8 hours by CFU scoring (S7A Fig) or OD_600_ measurement (S7B Fig) and compared it to the values obtained for the Δ*varA csrA*-Tn mutants (with WT and Δ*varA* as controls). The findings confirmed our assumption (S7 Fig) of the Δ*varA*Δ*aspA* mutant’s growth defect. Notably, an *aspA* single mutant grew indistinguishable from WT (S8 Fig).

We next looked into how exactly AspA overproduction might affect morphology. AspA is responsible for the reversible conversion of aspartate into ammonia and fumarate [34]. It is known that in many bacteria—including *V*. *cholerae*—L-aspartate is required for m-DAP synthesis as part of the lysine biosynthesis pathway (see KEGG online platform [35] accession number vch00300 for lysine biosynthesis in the *V*. *cholerae* reference strain N16961). Interestingly, recent work on the gram-positive bacterium *Bacillus subtilis* showed that an aspartate auxotroph mutant led to m-DAP depletion resulting in severe cell morphological defects such as bulging and, ultimately, cell lysis under specific growth conditions (e.g., Difco sporulation medium; [36]). While we did not observe significant cell lysis for the Δ*varA* cells, we still hypothesized that overproduction of AspA sequesters L-aspartate and, consequently, prevents m-DAP synthesis. To test this idea, we supplemented *varA-*deficient cells with L-aspartate. This significantly reduced cell rounding but did not alter the WT morphology (Fig 5E), indicating that L-aspartate is indeed sequestered by AspA.

**Fig 6:**
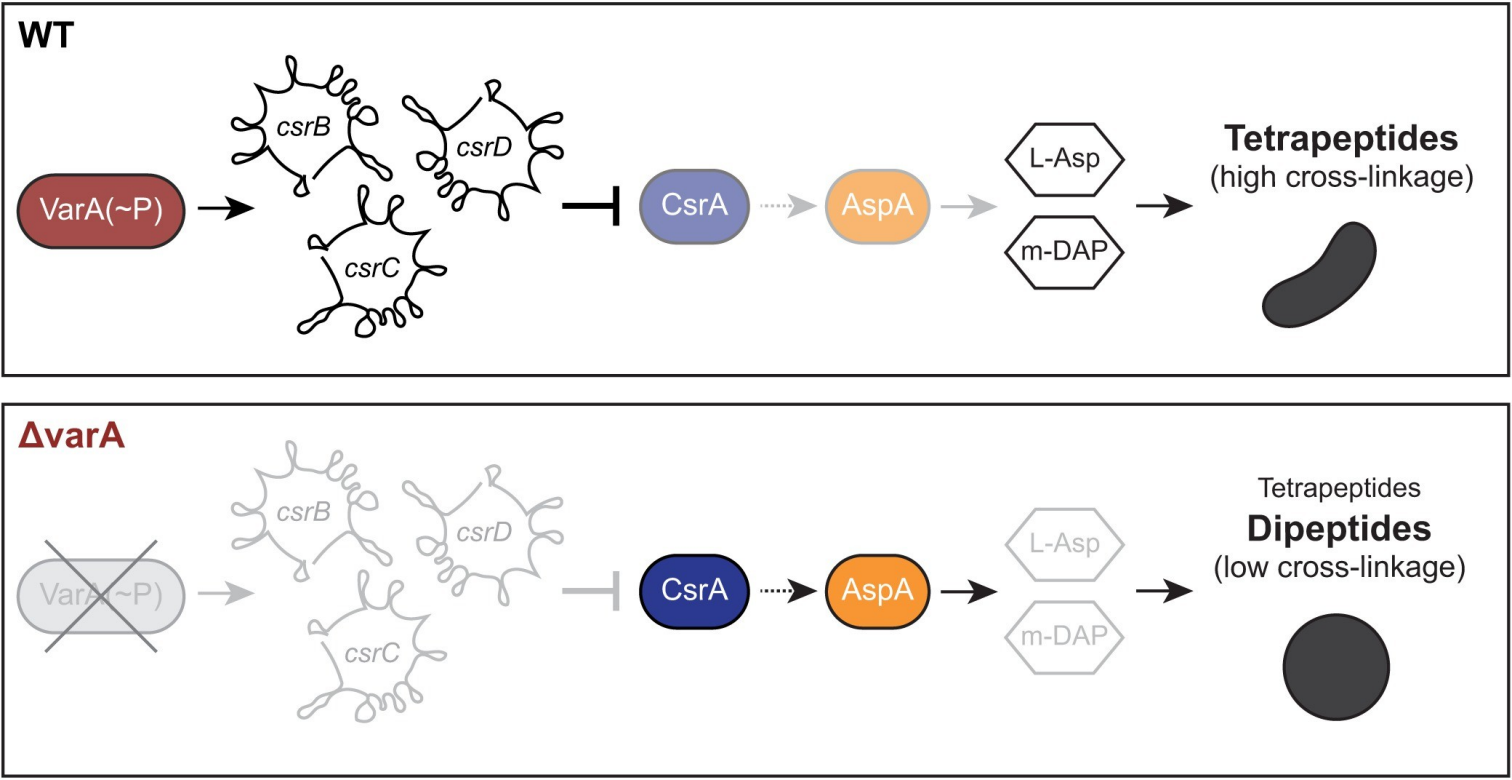
Model of VarA-CsrA signaling and impact on cell shape. For the WT, phosphorylated VarA [VarA(~P)] promotes the transcription of the sRNAs *csrB-D*, which sequesters CsrA. L-Asp and m-DAP levels are maintained resulting in normal PG and cell shape. In the absence of *varA*, the lack of the scavenging sRNAs results in increased CsrA activity and (directly or indirectly, as shown by the dashed arrow) overproduced AspA, which reduces L-Asp levels and impairs m-DAP biosynthesis. The PG therefore contains dipeptides, while tetrapeptides are reduced, leading to a reduction of cross-links and therefore cell rounding.

Taken all together, we propose a model whereby specific sequestering sRNAs are absent, causing an increased CsrA activity and the consequent overproduction of AspA, which reduces aspartate levels in Δ*varA* cells (Fig 6). These reduced aspartate levels subsequently impair the biosynthesis of the cell wall precursor m-DAP, thereby resulting in an abnormal dipeptide-containing under-crosslinked PG, which, ultimately, causes cell rounding (Fig 6). A caveat of our work and those of other researchers working on this pathway is, however, that the phenotype was solely observed in a genetic knock-out while we still miss the physiological conditions that reflect such *varA* deficiency. Notably, the signal(s) that abrogate(s) VarA phosphorylation by VarS, or any other additional histidine kinases, have not been unambiguously identified. Such conditions are expected to mimic the *varA*-deficient phenotypes that have been described in several previous studies [9–13], which we complement here with our finding of a VarA/CsrA-dependent PG modification and cell shape alteration. Thereupon, future studies are required to identify the conditions that alter VarA signaling during human infection and/or growth of *V*. *cholerae* in its natural aquatic habitat.

Our study shows that impairment of the VarA-CsrA signaling pathway in *V*. *cholerae* leads to a modulation of bacterial morphology during the stationary growth phase by altering the synthesis of cell wall precursors. While the advantage of this alteration is so far unknown for *V*. *cholerae*, such cell shape transitions have been demonstrated for other pathogens, such as *C*. *jejuni* and *H*. *pylori* [22,23]. Moreover, growth phase-dependent cell wall remodeling occurs in many bacteria. For instance, during stationary phase, *V*. *cholerae* cells synthesize dedicated D-amino acids (D-aa), which are incorporated into their PG mesh [37]. Production and insertion of such D-aa was hypothesized to be a strategy for adapting to changing conditions. Consistent with this idea, Le and colleagues recently demonstrated the incorporation of non-canonical D-aa into the PG of *Acinetobacter baumannii* during stationary phase [38]. Interestingly, this PG editing process protected the pathogen from PG-targeting type VI secretion system-dependent effector proteins [38]. Therefore, we postulate that round-shaped *V*. *cholerae* might encounter similar fitness benefits during interbacterial competition, antibiotic treatment, or other, so far unidentified, conditions.

Overall, our work has deciphered a new role of the VarA-CsrA signaling pathway in cell shape transition through the modulation of cell wall precursor synthesis. Due to the role of this pathway in bacterial virulence, understanding its purpose in cellular adaptation and survival is a key component of understanding bacterial changes that lead to human infections. The future studies we envision should therefore aim at identifying the conditions that lead to this altered signaling and conferred fitness benefits.

## Materials and methods

### Bacterial strains and plasmids

The bacterial strains and plasmids used in this work are provided in S4 and S5 Tables, respectively. The primary *V*. *cholerae* isolate used throughout this study is O1 El Tor strain A1552 [39], a representative of the ongoing 7^th^ cholera pandemic. Genetic manipulations are based on its published genome sequence [29].

### Growth conditions and medium supplementation

Bacterial cultures were grown aerobically with agitation (180 rpm) at 30°C and 37°C for *Vibrio* spp. and *E*. *coli*, respectively. As for the liquid medium, home-made lysogeny broth (10 g/L Tryptone, AppliChem; 10 g/L NaCl, Fisher scientific; 5 g/L Yeast Extract, AppliChem) was used or, when explicitly mentioned, a variation without NaCl (LB_0_). LB-agar (including 1.5% agar; Carl Roth) was used as a solid medium. All liquid cultures were grown overnight prior to being back-diluted (1:100) into fresh LB and were cultured for the indicated duration. When required, LB cultures were supplemented at 3h post-dilution with 7.5 mM L-aspartate (Sigma-Aldrich). Growth was monitored by measuring the optical density at 600 nm (OD_600_) and by enumerating the colony forming units (CFU) after serial dilution.

The conditioned medium was obtained from cultures grown for 8 h through centrifugation (4,000 rpm for 15 min at room temperature [RT]) and subsequent filter sterilization (0.2 μm filter) and were stored at 4°C until the next day. Prior to usage, the conditioned medium was diluted 1:1 in two-fold concentrated LB medium (2×LB).

For natural transformation assays on chitin flakes, 0.5× defined artificial sea water (DASW) supplemented with 50 mM HEPES (Sigma-Aldrich) and vitamins (MEM, Gibco) was used, as described in [40]. Thiosulfate citrate bile salts sucrose (TCBS; Sigma-Aldrich) agar plates were used to counter-select *E*. *coli* after bi-/or tri-parental mating. NaCl-free LB plates supplemented with 10% sucrose (Sigma-Aldrich) were used for the *sacB*-based counter-selection.

Whenever required, antibiotics were added at the following concentrations: ampicillin (Amp; 100 μg/ml), gentamicin (Gent; 50 μg/ml), kanamycin (Kan; 75 μg/ml), and rifampicin (Rif; 100 μg/ml).

### Genetic engineering of strains and plasmid constructions

Standard molecular biology-based techniques were used for molecular cloning [41]. All genetically engineered strains were verified by PCR and confirmed by Sanger sequencing of the modified genomic regions (Microsynth AG, Switzerland). Deletion mutants of *V*. *cholerae* were generated via either an allelic exchange approach using the counter-selectable plasmid pGP704-Sac28 [42] or via a combination of natural transformation and Flip recombination to remove the selection cassette [TransFLP; [43–45]]. Tri-parental mating was used to site-directly integrate the mini-Tn7 transposon into the *V*. *cholerae* large chromosome [46]. For complementation purposes, the transposon carried the gene-of-interest preceded by its promotor-containing upstream region.

### Light microscopy imaging

Thin agarose pads (1.2% in 0.5×PBS) were used to coat microscope slides. Bacteria were immobilized by mounting them on top of the pads, which were then covered with a coverslip. The cells were observed using a Zeiss Axio Imager M2 epifluorescence microscope, controlled by the Zeiss Zen 2.6 software (blue edition). All images were analyzed and prepared for publication using Fiji [47]. For shape quantification purposes, single cell analysis was performed using MicrobeJ [15]. For the latter approach, at least 10 images were taken per condition, from which 1000 bacterial cells were randomly selected for the final quantification. The cells’ volume was estimated using the measured length (l) and width (w) values obtained with MicrobeJ [15] in the following equation: volume = $\left(l*{\pi *(\frac{w}{2})}^{2}\right)-\frac{1}{3}\left(2\pi *{\left(\frac{w}{2}\right)}^{3}\right)$.

### Time-lapse microscopy imaging

For time-lapse microscopy experiments, the bacteria were pre-grown for 20h as described above. At that time, the cells were back-diluted (1:100) into fresh LB medium and loaded into a 35mm μ-Dish (Ibidi, Martinsried, Germany). The bacteria were further incubated at room temperature inside the dish, which was installed in the microscope. Images were taken every 5 min for 2.5 hours using an inverted Zeiss LSM700 confocal microscope equipped with a fluorescence light source (Illuminator HXP 120), an AxioCam MRm high resolution camera, and controlled by the Zeiss Zen 2012 software (blue edition).

### Cryo-electron microscopy

Restreaked colonies on LB-agar were used to inoculate LB medium to grow bacteria overnight. The resulting cultures were then back-diluted (1:100) into fresh LB medium. At 20 h post-dilution, an aliquot was removed, mixed with 15 nm gold beads (Cell Microscopy Core, Utrecht University, Utrecht, The Netherlands), loaded onto a R2/2 200 mesh Cu grid (Quantifoil Micro Tools, GmbH), and plunge frozen in liquid ethane using the automated Leica EM GP plunge freezer (Leica Microsystems GmbH). Vitrified cells were imaged using a Gatan 626 cryoholder using a Talos L120C electron microscope (Thermo Fisher Scientific, TFS). Images were acquired with a Ceta CMOS camera (TFS) with a magnification range of 1,600–17,500×, corresponding to a pixel size of 6.29–0.584 nm/pixel (FOV 25.16–2.336 μm^2^).

### Peptidoglycan analysis

PG was isolated from at least two biological replicates and analyzed by High Performance Liquid Chromatography (HPLC), as previously described [48]. Cells were grown at 30°C in 400 mL of LB medium with agitation for 2 h and 20 h. Cells were cooled on ice for 15 min and were collected by centrifugation for 15 min at 4°C and 3,220 × *g*. Cell pellets were resuspended in 6 mL of cold water and lysed by adding them dropwise to 6 mL of boiling 8% SDS (Sigma-Aldrich) within 10 min, under vigorous stirring. Samples were boiled for an additional 30 min and subsequently cooled to RT and stored until further analysis.

Crude PG was collected by centrifugation for 60 min at 90,000 × *g* at 28°C. Pellets were washed several times with warm water until the SDS was removed. Samples were treated with α-amylase and pronase to remove high–molecular weight glycogen and PG-associated proteins, respectively. The resulting PG was boiled in 4% SDS and was washed free of SDS as described [48].

The PG composition (muropeptide profile) was analyzed as previously described [48]. Briefly, muropeptides were generated from PG using the muramidase cellosyl (Hoechst, Frankfurt am Main, Germany). The reaction was stopped by heating the samples at 100°C for 10 min and the sample was centrifuged for 10 min at 13,000 × *g* to clarify the solution. Muropeptides present in the supernatant were reduced with sodium borohydride and analyzed by HPLC using a 250 × 4.6 mm, 3 μm ProntoSIL 120-3-6C18 AQ reversed phase column (Bischoff, Leonberg, Germany) on an Agilent 1100 system. The eluted muropeptides were detected by their absorbance at 205 nm. *V*. *cholerae* muropeptides were assigned according to their known retention times and quantified by their peak area using the Laura software (Lab Logic Systems). The muropeptide fraction 4 was collected and analyzed by mass spectrometry at the Newcastle University Pinnacle facility as described previously [49].

### Transposon mutagenesis screen

Transposon insertion libraries were prepared in the ΔvarA strain by introducing the mariner-based transposon (Kan^R^) carried on plasmid pSC189 [50] via conjugation, as previously described [51]. Following growth at 30°C overnight, colonies (~100,000) were scrapped from the plates and resuspended in PBS buffer. To screen for mutants resistant to osmotic stress, the library was diluted 1:100 in LB_0_ plus kanamycin and was incubated at 30°C for 1 h with agitation. Surviving bacteria were concentrated by centrifugation (3,220 × *g* for 15 min at RT), resuspended in regular LB_10_ medium (containing kanamycin) and cultured at 30°C overnight. Following two additional rounds of selection in LB_0_ medium, the library was stored in LB medium containing 20% glycerol at -80°C until further analysis.

Next, the libraries were thawed, mixed with LB_10_ + Kan, and grown at 30°C overnight before being back-diluted 1:100 in fresh medium and grown for approximately 20 h. After visualization of the bacterial cell morphology by microscopy to confirm the absence of round cells, the resulting cultures were streaked on LB-agar plates (containing kanamycin) to obtain single colonies. To identify transposon insertion sites, 24 colonies were randomly picked and subjected to two-step arbitrary PCR, followed by Sanger sequencing to identify the transposition site, as previously described [51].

### SDS-PAGE, Western blotting, and Coomassie blue staining

Bacteria were grown as described above until they reached an OD_600_ of approximately 2.5. At that point, the cultures were harvested by centrifugation (20,000 × *g* for 3 min at RT) and the pellet was resuspended in a volume of 2× Laemmli buffer (Sigma-Aldrich) that adjusts for the total number of bacteria according to the OD_600_ of the initial cultures followed by 15 min incubation at 95°C. The proteins were resolved by SDS-PAGE using 8–16% Mini-PROTEAN TGX Stain-Free protein gels (Bio-Rad) and transferred onto a PVDF membrane using a semi-dry apparatus (Trans-Blot Turbo Transfer System; Bio-Rad). The detection of the signal from the HapR protein or Sigma70 as a loading control was performed as described [51]. Densitometry band quantification was done using Fiji [47]. The relative abundance values for HapR were normalized to the σ^70^ loading control and to the WT (A1552) as the positive control.

Coomassie blue staining was performed after SDS-PAGE to identify under- or overproduced proteins. To do so, the gels were soaked in the Coomassie blue solution (0.2% Coomassie brilliant blue [A1092; AppliChem] in 10% methanol plus 1% acetic acid) and stained for 30 min at RT with gentle agitation. Three destaining steps (30 min each) were performed using a destaining solution (10% methanol plus 1% acetic acid).

### Protein identification through mass spectrometry

The region of interest (e.g., with the overproduced band in the Δ*varA* strain) was cut from a polyacrylamide gel previously stained with Coomassie blue as described above. The gel pieces (from the WT and Δ*varA* samples for comparison reason) were washed twice in 50% ethanol and 50 mM ammonium bicarbonate (Sigma-Aldrich) for 20 min and dried by vacuum centrifugation. Sample reduction was performed with 10 mM dithioerythritol (Merck-Millipore) for 1 h at 56°C. This washing-drying step was repeated before performing the alkylation step with 55 mM iodoacetamide (Sigma-Aldrich) for 45 min at 37°C in the dark. Next, samples were washed-dried once again and digested overnight at 37°C using mass spectrometry grade Trypsin gold (Trypsin Gold, Promega) at a concentration of 12.5 ng/μl in 50 mM ammonium bicarbonate and 10 mM CaCl_2_. The resulting peptides were extracted in 70% ethanol plus 5% formic acid (Merck-Millipore) twice for 20 min with permanent shaking. Samples were further dried by vacuum centrifugation and stored at -20°C. Peptides were desalted on C18 StageTips [52] and dried by vacuum centrifugation prior to LC-MS/MS injections. Samples were resuspended in 2% acetonitrile (Biosolve) and 0.1% formic acid, and nano-flow separations were performed on a Dionex Ultimate 3000 RSLC nano UPLC system (Thermo Fischer Scientific) online connected with a Q Exactive HF Orbitrap mass spectrometer (Thermo Fischer Scientific). A capillary precolumn (Acclaim Pepmap C18, 3 μm-100 Å, 2 cm × 75 μm ID) was used for sample trapping and cleaning. A 50 cm long capillary column (75 μm ID; in-house packed using ReproSil-Pur C18-AQ 1.9 μm silica beads; Dr. Maisch) was then used for analytical separations at 250 nl/min over 90 min biphasic gradients. Acquisitions were performed through Data-Dependent Acquisition (DDA). First MS scans were acquired with a resolution of 60,000 (at 200 m/z) and the 15 most intense parent ions were then selected and fragmented by High energy Collision Dissociation (HCD) with a Normalized Collision Energy (NCE) of 27% using an isolation window of 1.4m/z. Fragmented ions were acquired with a resolution of 15,000 (at 200m/z) and selected ions were then excluded for the following 20 s. Raw data were processed using SEQUEST in Proteome Discoverer v.2.2 against a home-made database (3610 entries) using the genome sequence of *V*. *cholerae* strain A1552 as an input (GenBank accession numbers CP028894 for chromosome 1 and CP028895 for chromosome 2) [29]. Enzyme specificity was set to trypsin and a minimum of six amino acids were required for peptide identification. Up to two missed cleavages were allowed. A 1% FDR cut-off was applied both at peptide and protein identification levels. For the database search, carbamidomethylation was set as a fixed modification, whereas oxidation (M), acetylation (protein N-term), PyroGlu (N-term Q), and Phosphorylation (S,T,Y) were considered as variable modifications. Data were further processed and inspected in Scaffold 4.10 (Proteome Software, Portland, USA).

### Quantitative reverse transcription PCR (qRT-PCR)

Bacterial cultures were grown until they reached an OD_600_ of approximately 2.5, as described above. The purification of RNA, DNase treatment, cDNA synthesis, and qPCR were performed according to a previously reported protocol [53]. qPCR runs were performed on a LightCycler Nano or LightCycler 96 Real-Time PCR System (Roche). The data was analyzed with the respective Roche software packages using the standard curve method. Expression values in the graphs are based on three independent experiments and presented as relative to the mRNA levels of the housekeeping gene *gyrA*.

### RNA sequencing (RNAseq)

Expression profiling by RNA sequencing was performed as previously described [54]. Briefly, bacteria were grown as described above until they reached an OD_600_ of approximately 0.2 and 2.5. At that time, cultures were harvested by centrifugation (20,000 x g for 3 min at 4°C) and the pellet washed with pre-chilled PBS buffer. Cells were lysed through resuspension in Tri reagent (Sigma) followed by shock freezing in a dry-ice ethanol bath. Until processing, the samples were stored at -80°C. The preparation of RNA and DNase treatment were performed as previously reported [53]. The samples were re-purified after DNase treatment using a supplementary RNA clean-up procedure with the GenElute Mammalian Total RNA miniprep kit (Sigma). Further processing of the samples, sequencing, and the data analysis was conducted by Microsynth AG (Balgach, Switzerland) as recently described [55]. Three biologically independent experiments were performed.

## Supporting information

S1 Fig *V*. *cholerae* strains lacking VarA but not VarS display an atypical round shape late during growth. Phase contrast micrographs (top) and roundness quantification (bottom) of the WT, Δ*varA*, Δ*varS*, complemented Δ*varA*+*varA*, and Δ*varS*Δ*varA* strains. Cells were imaged at 20 h post-dilution. Scale bar: 2 μm. The roundness quantification is based on 3000 cells (n = 1000 per independent repeat) using the MicrobeJ software.
(TIF)

S2 Fig Rounded *varA*-deficient cells have an increased volume. Estimated cell volume distributions for the WT and Δ*varA* strain. Cells were imaged at 20 h post-dilution and the volume was estimated based on the length and width of the cells using the MicrobeJ software. The values are based on 3000 cells for each strain (n = 1000 per independent experiment).
(TIF)

S3 Fig The Δ*varS* and Δ*varA* strains differ in their *csrB*, *csrC*, and *csrD* expression. Relative expression values for *csrB*, *csrC*, and *csrD* for the WT, Δ*varA*, and Δ*varS* strains sampled at an OD_600_ of ~2.5. The bars are based on the mean of the three independent experiments (with circles showing the individual experiments) and the error bars show the S.D. Statistics was performed on log-transformed data and based on a two-way ANOVA with Dunnett’s multiple comparisons tests, in which each strain was compared to the WT. *, *P* < 0.05; ****, *P* < 0.0001; n.s., not significant.
(TIF)

S4 Fig The Δ*varA* mutant has a slight growth defect. Growth curves and final yields for the WT and Δ*varA* strains. (A and B) Enumeration of colony forming units (CFU) and (B and C) optical density measurements at 600 nm (OD_600_) were performed every hour for 8 h (A and C) and at 24 h post-dilution (B and D). Each value represents the mean of three independent experiments (± S.D., as shown by the error bars). Statistical analyses (B, log-transformed and D) were based on unpaired *t* tests with Welch’s correction. n.s., not significant.
(TIF)

S5 Fig The Δ*varA* mutant is QS-proficient. (A and B) HapR is produced and active in Δ*varA* strains. Phase contrast micrographs of the following strains after growth for 20 h: Δ*hapR* and Δ*hapR*Δ*varA* strains (A; scale bar: 5 μm) or WT, Δ*varA*, Δ*luxO*, and Δ*luxO*Δ*varA* (B; scale bar: 2 μm). Roundness quantification of n = 3000 cells for each condition is provided in (B). (C) QS-impaired LuxO* variants abrogate HapR production. Detection of HapR by western blotting for WT and luxO* variants of strains A1552 and C6706 in the presence or absence of *varA*. All strains were sampled at OD_600_ ~ 2.5. Representative blots from three independent experiments. The relative abundance of HapR protein is normalized to the σ^70^ loading control and to the WT (A1552), which was set to 100.
(TIF)

S6 Fig Δ*varA* cells do not show periplasmic or membrane defects. Ultrastructural analysis of WT and Δ*varA* cells imaged by cryo-electron microscopy. Representative images from three independent experiments. The cells were sampled at 20 h post-dilution. Scale bar: 500 nm.
(TIF)

S7 Fig Growth phenotypes of defined and suppressor mutants in the Δ*varA* background. Growth phenotypes of the WT, Δ*varA*, Δ*aspA*Δ*varA*, and selected Δ*varA*-Tn suppressor mutants (mutant A, C, and H). (A) Enumeration of colony forming units (CFU) or (B) optical density measurements at 600 nm (OD_600_) were performed every hour for 8 h post-dilution. Each value corresponds to the mean of three independent experiments (± S.D.).
(TIF)

S8 Fig The *aspA* deletion itself has no impact on bacterial growth. Growth curves of the WT, Δ*varA*, Δ*aspA*, Δ*aspA*Δ*varA*, and Δ*aspA*Δ*varA*+*aspA* strains. (A and B) The colony forming units (CFU) and (B and C) the optical density at 600 nm (OD_600_) were measured every hour for 8 h (A and C) and again after 24 h post-dilution (B and D). Bars represent the average of three independent experiments and the error bars correspond to the S.D.. Statistical analyses (B, log-transformed and D) are based on one-way ANOVA with Dunnett’s multiple comparisons tests whereby each mutant is compared to the WT. n.s., not significant.
(TIF)

S1 Movie Round Δ*varA* cells regain WT shape after dilution in fresh medium. Representative movie showing the growth and division of Δ*varA* cells after dilution in fresh medium. Images were taken every 5min for 2.5 hours. Scale bar: 10 μm.
(AVI)

S1 Table Summary of the muropeptide composition of diverse *V*. *cholerae* strains. (DOCX)

S2 Table Summary of the muropeptide composition of shape-restored mutants in the Δ*varA* background. (DOCX)

S3 Table RNAseq data showing normalized reads for each locus tag of strain A1552. (XLSX)

S4 Table Bacterial strains used in this study. (DOCX)

S5 Table Plasmids used in this study. (DOCX)

S1 Data Numerical data for all figures. (XLSX)
