# Supplementary figures and images for: The VarA-CsrA regulatory pathway influences cell shape in Vibrio cholerae
Source: PLoS Genet. 2022 Mar 28;18(3):e1010143. doi: 10.1371/journal.pgen.1010143 (PMC8989286; doi:10.1371/journal.pgen.1010143)

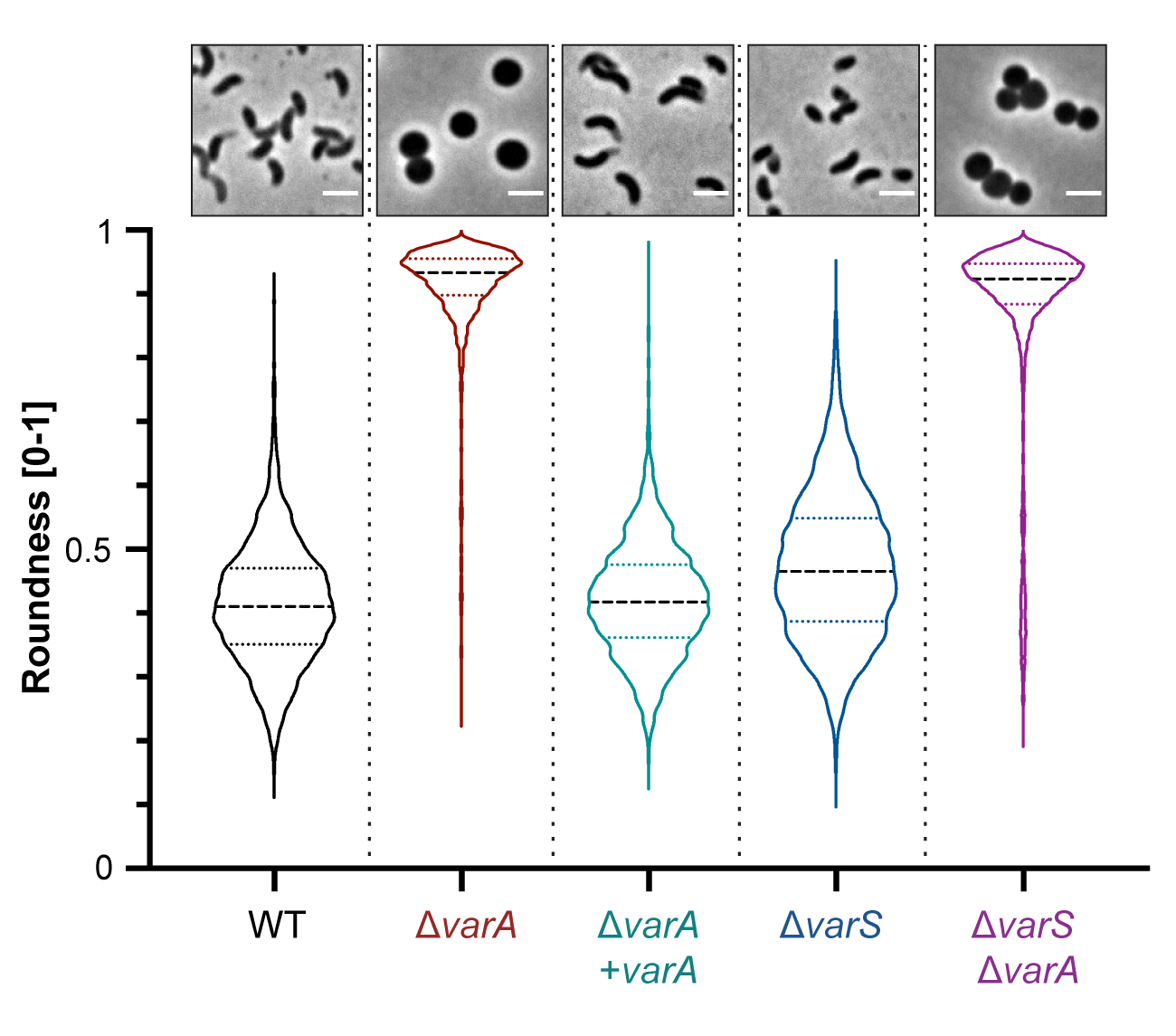

Supplement: S1 Fig — Phase contrast micrographs (top) and roundness quantification (bottom) of the WT, ΔvarA, ΔvarS, complemented ΔvarA+varA, and ΔvarSΔvarA strains. Cells were imaged at 20 h post-dilution. Scale bar: 2 μm. The roundness quantification is based on 3000 cells (n = 1000 per independent repeat) using the MicrobeJ software. (TIF) [file pgen.1010143.s001.tif]

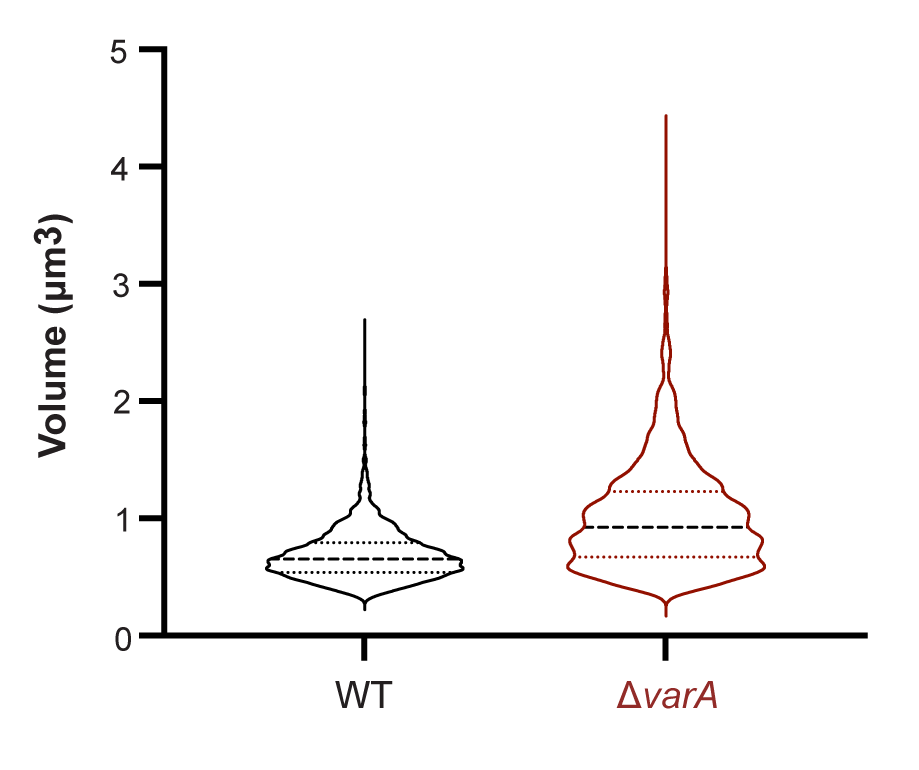

Supplement: S2 Fig — Estimated cell volume distributions for the WT and ΔvarA strain. Cells were imaged at 20 h post-dilution and the volume was estimated based on the length and width of the cells using the MicrobeJ software. The values are based on 3000 cells for each strain (n = 1000 per independent experiment). (TIF) [file pgen.1010143.s002.tif]

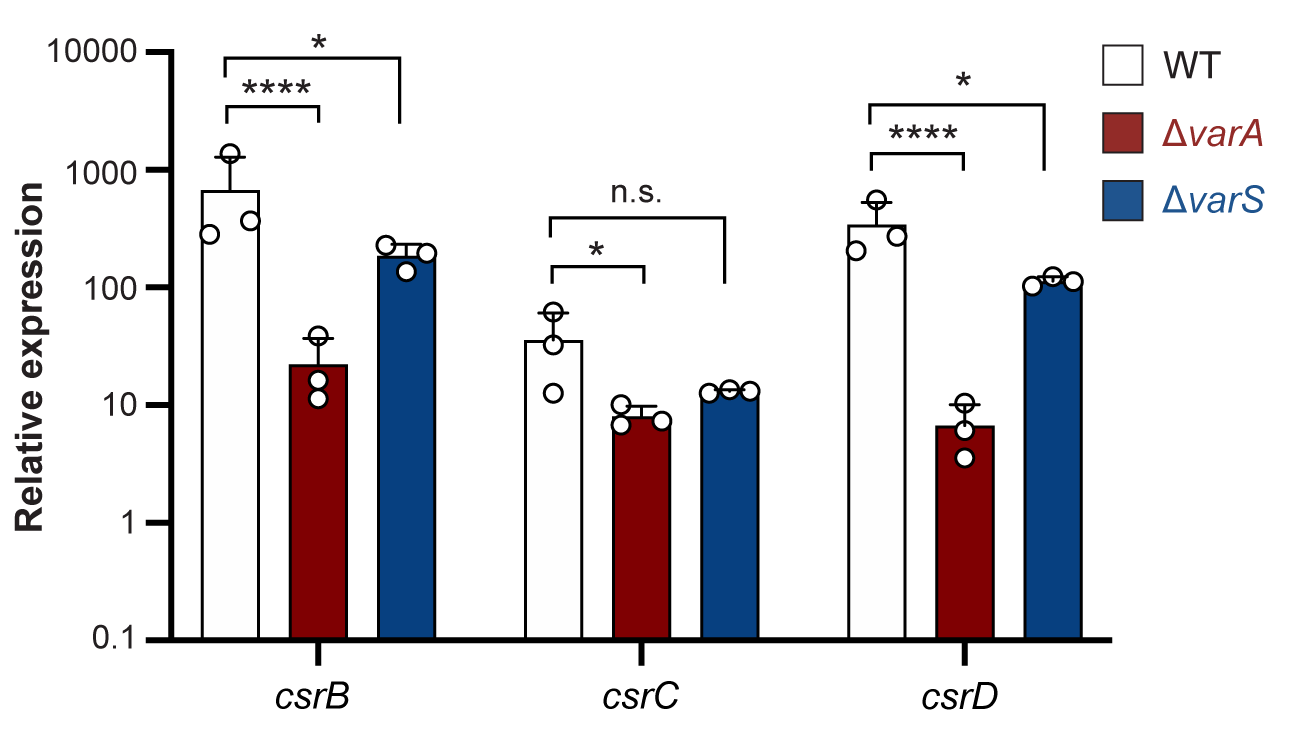

Supplement: S3 Fig — Relative expression values for csrB, csrC, and csrD for the WT, ΔvarA, and ΔvarS strains sampled at an OD600 of ~2.5. The bars are based on the mean of the three independent experiments (with circles showing the individual experiments) and the error bars show the S.D. Statistics was performed on log-transformed data and based on a two-way ANOVA with Dunnett’s multiple comparisons tests, in which each strain was compared to the WT. *, P < 0.05; ****, P < 0.0001; n.s., not significant. (TIF) [file pgen.1010143.s003.tif]

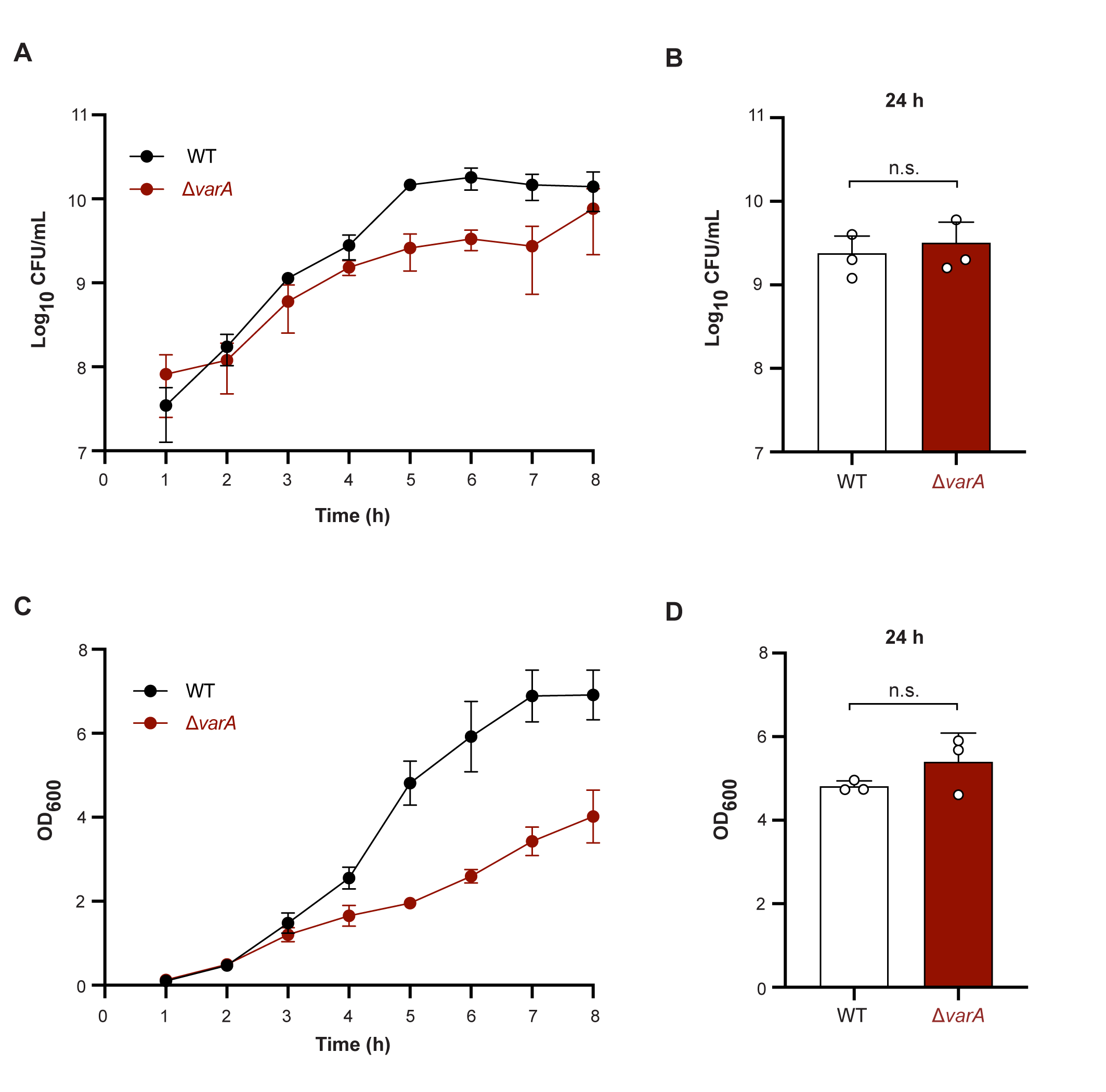

Supplement: S4 Fig — Growth curves and final yields for the WT and ΔvarA strains. (A and B) Enumeration of colony forming units (CFU) and (B and C) optical density measurements at 600 nm (OD600) were performed every hour for 8 h (A and C) and at 24 h post-dilution (B and D). Each value represents the mean of three independent experiments (± S.D., as shown by the error bars). Statistical analyses (B, log-transformed and D) were based on unpaired t tests with Welch’s correction. n.s., not significant. (TIF) [file pgen.1010143.s004.tif]

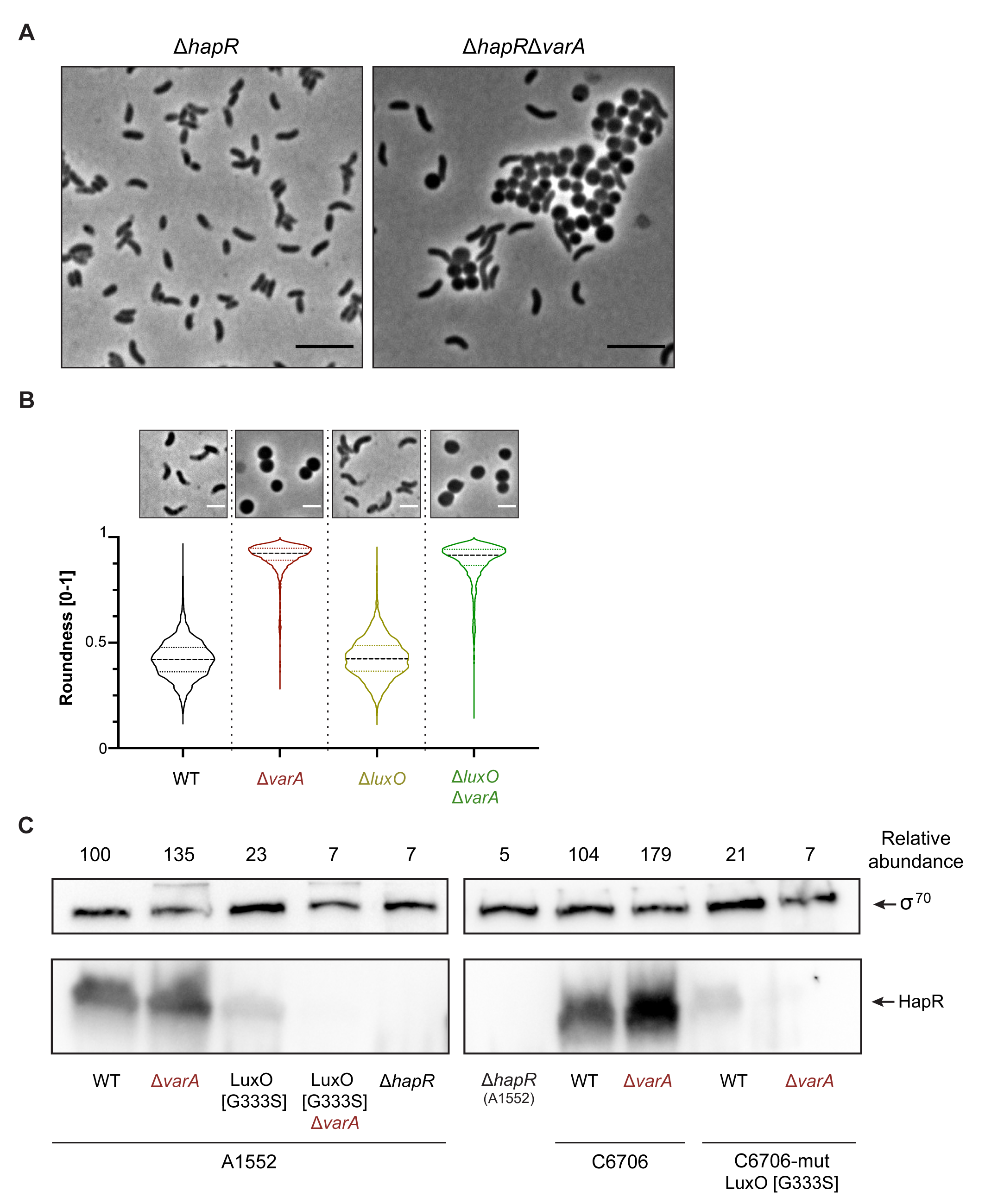

Supplement: S5 Fig — (A and B) HapR is produced and active in ΔvarA strains. Phase contrast micrographs of the following strains after growth for 20 h: ΔhapR and ΔhapRΔvarA strains (A; scale bar: 5 μm) or WT, ΔvarA, ΔluxO, and ΔluxOΔvarA (B; scale bar: 2 μm). Roundness quantification of n = 3000 cells for each condition is provided in (B). (C) QS-impaired LuxO* variants abrogate HapR production. Detection of HapR by western blotting for WT and luxO* variants of strains A1552 and C6706 in the presence or absence of varA. All strains were sampled at OD600 ~ 2.5. Representative blots from three independent experiments. The relative abundance of HapR protein is normalized to the σ70 loading control and to the WT (A1552), which was set to 100. (TIF) [file pgen.1010143.s005.tif]

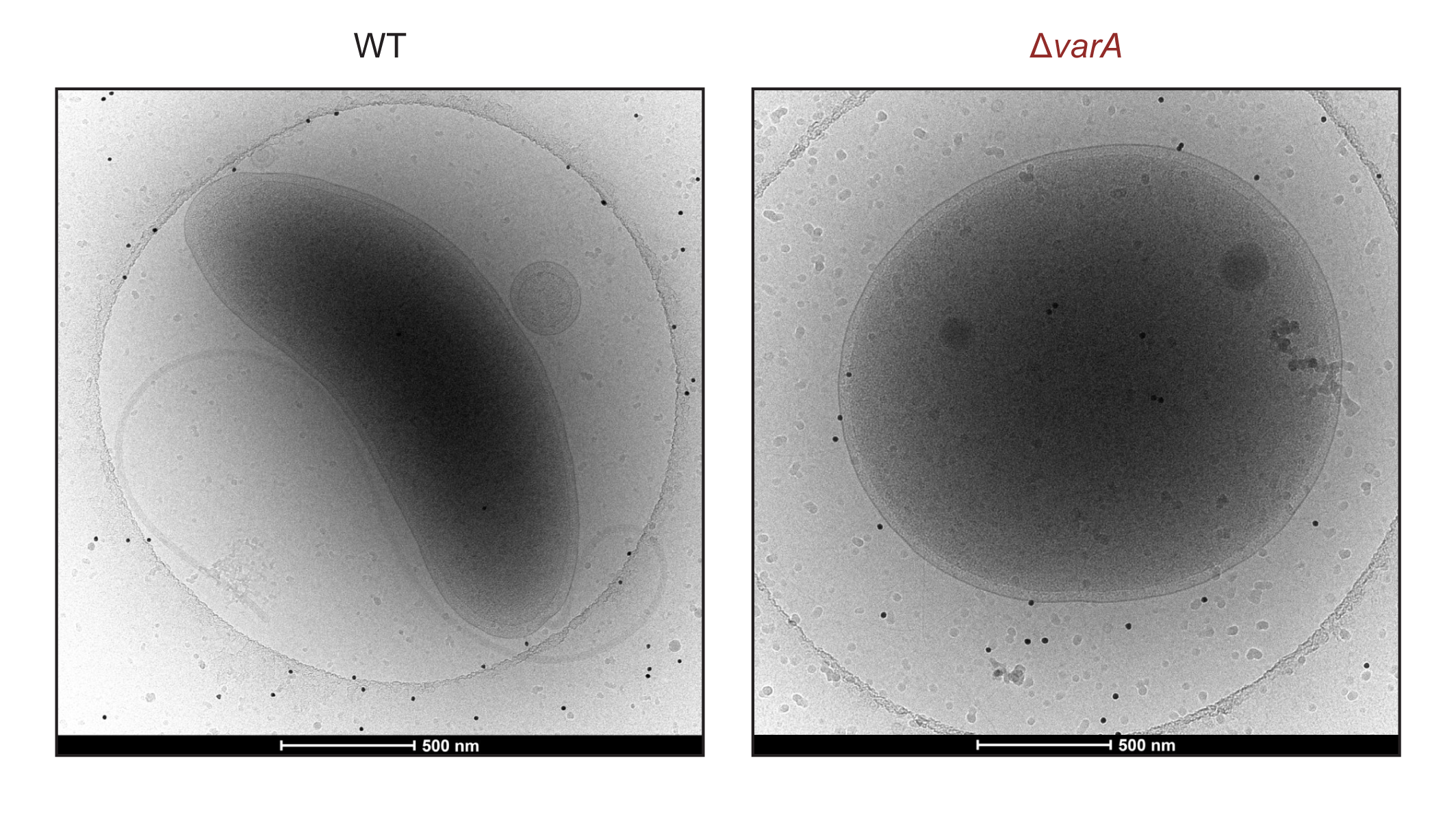

Supplement: S6 Fig — Ultrastructural analysis of WT and ΔvarA cells imaged by cryo-electron microscopy. Representative images from three independent experiments. The cells were sampled at 20 h post-dilution. Scale bar: 500 nm. (TIF) [file pgen.1010143.s006.tif]

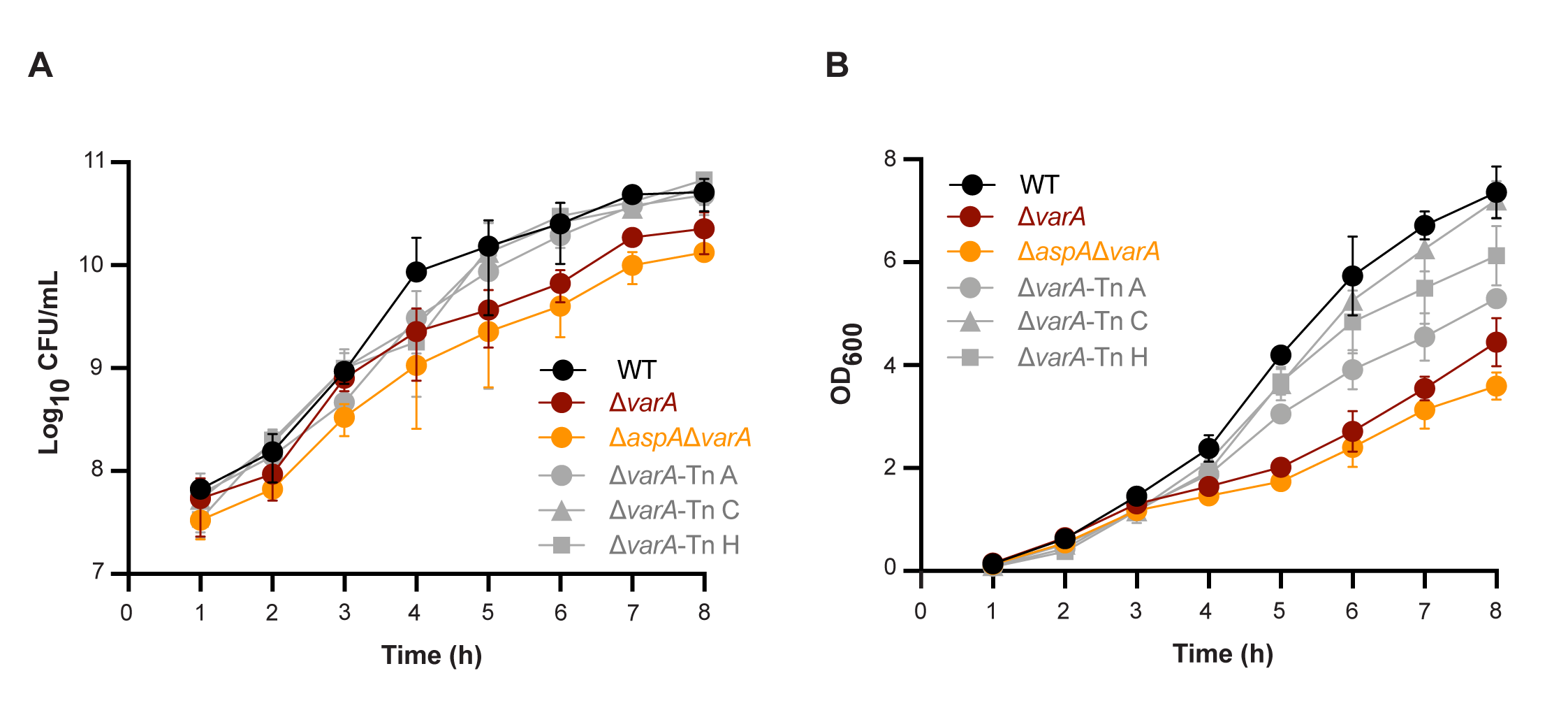

Supplement: S7 Fig — Growth phenotypes of the WT, ΔvarA, ΔaspAΔvarA, and selected ΔvarA-Tn suppressor mutants (mutant A, C, and H). (A) Enumeration of colony forming units (CFU) or (B) optical density measurements at 600 nm (OD600) were performed every hour for 8 h post-dilution. Each value corresponds to the mean of three independent experiments (± S.D.). (TIF) [file pgen.1010143.s007.tif]

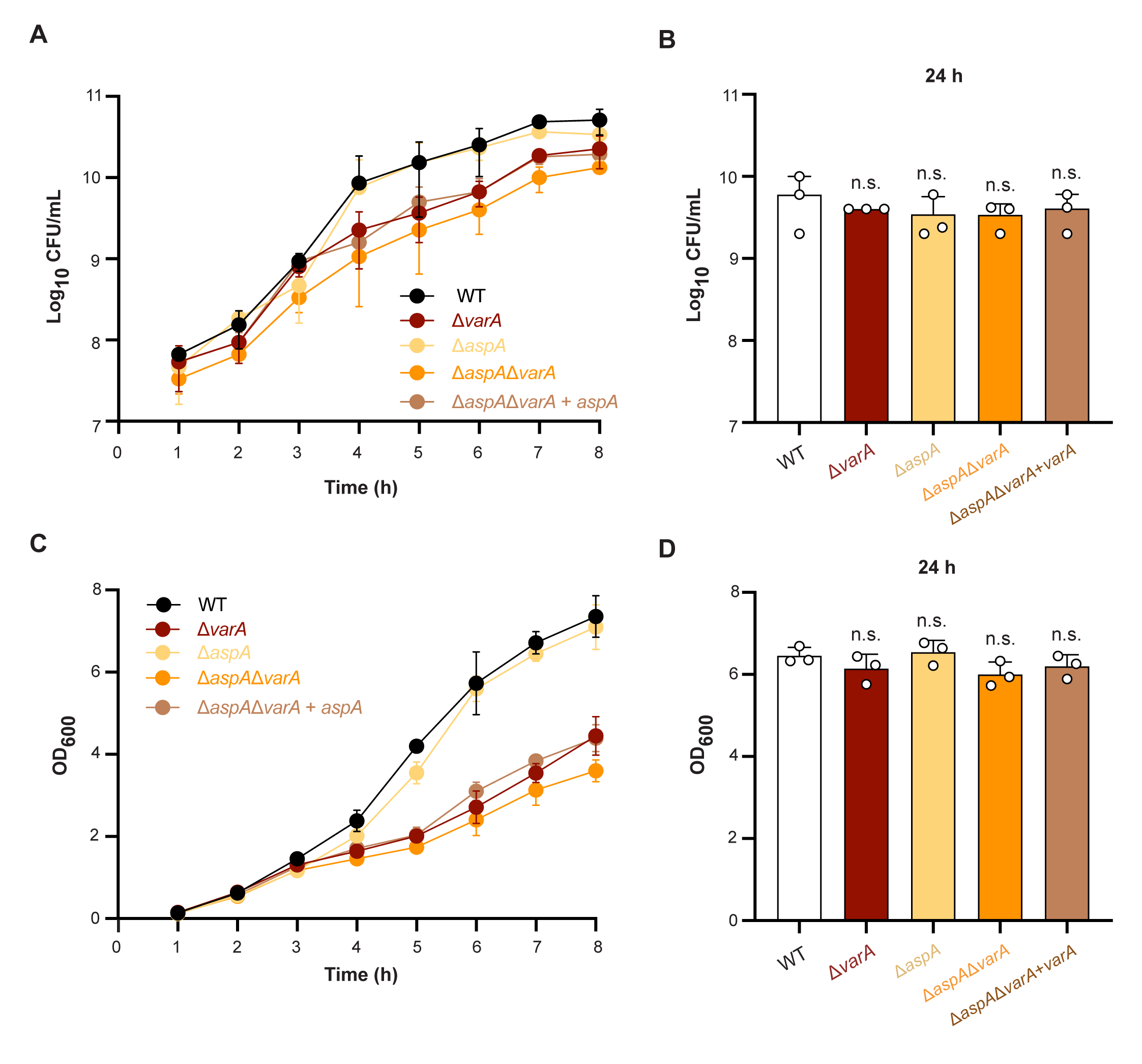

Supplement: S8 Fig — Growth curves of the WT, ΔvarA, ΔaspA, ΔaspAΔvarA, and ΔaspAΔvarA+aspA strains. (A and B) The colony forming units (CFU) and (B and C) the optical density at 600 nm (OD600) were measured every hour for 8 h (A and C) and again after 24 h post-dilution (B and D). Bars represent the average of three independent experiments and the error bars correspond to the S.D.. Statistical analyses (B, log-transformed and D) are based on one-way ANOVA with Dunnett’s multiple comparisons tests whereby each mutant is compared to the WT. n.s., not significant. (TIF) [file pgen.1010143.s008.tif]
