# Supplementary material for: The VarA-CsrA regulatory pathway influences cell shape in Vibrio cholerae
Source: PLoS Genet. 2022 Mar 28;18(3):e1010143. doi: 10.1371/journal.pgen.1010143 (PMC8989286; doi:10.1371/journal.pgen.1010143)
Supplement: S1 Table — (DOCX) [file pgen.1010143.s010.docx]

| **Muropeptide^2^** | **Relative % of muropeptide^1^** | | | | | |
| --- | --- | --- | --- | --- | --- | --- |
|  | **2 h post-dilution** | | | **20 h post-dilution** | | |
|  | **WT** | **Δ*varA*** | **Δ*varA***  **+*varA*** | **WT** | **Δ*varA*** | **Δ*varA***  **+*varA*** |
| **Tri** | 0.7 ± 0.2 | 1.6 ± 0.9 | 0.6 ± 0.0 | 2.0 ± 0.3 | 4.8 ± 0.4 | 3.0 ± 0.3 |
| **TetraGly4** | 1.2 ± 0.4 | 2.3 ± 1.1 | 0.8 ± 0.1 | 1.0 ± 0.2 | 1.2 ± 0.1 | 1.5 ± 0.0 |
| **Tetra** | 49.4 ± 1.0 | 46.1 ± 3.1 | 49.4 ± 0.8 | 42.6 ± 2.9 | 27.7 ± 1.1 | 40.4 ± 0.5 |
| **Di^3^** | 0.0 ± 0.0 | 1.7 ± 0.4 | 0.0 ± 0.0 | 0.0 ± 0.0 | 24.4 ± 0.0 | 0.0 ± 0.0 |
| **Penta** | 2.8 ± 0.4 | 2.2 ± 0.7 | 3.1 ± 0.5 | 0.6 ± 0.1 | 2.5 ± 0.4 | 0.8 ± 0.3 |
| ***unknown*** | 0.7 ± 0.1 | 1.4 ± 0.2 | 0.8 ± 0.1 | 2.0 ± 1.1 | 4.5 ± 0.9 | 2.4 ± 0.5 |
| **TetraTri(Dap)** | 0.6 ± 0.2 | 3.5 ± 2 | 0.4 ± 0.4 | 1.6 ± 0.3 | 3.4 ± 1.2 | 4.4 ± 2.6 |
| **TetraTri** | 0.3 ± 0.3 | 1.1 ± 0.2 | 0.3 ± 0.3 | 1.6 ± 0.3 | 2.5 ± 0.2 | 2.1 ± 0.2 |
| **TetraTetra** | 30.2 ± 0.0 | 27.4 ± 0.6 | 30.5 ± 0.5 | 29.0 ± 0.3 | 14.8 ± 0.6 | 27.8 ± 0.6 |
| **TetraPenta** | 1.0 ± 0.2 | 0.8 ± 0.0 | 1.2 ± 0.2 | 1.0 ± 0.5 | 1.3 ± 0.2 | 1.0 ± 0.0 |
| **TetraTetraTetra** | 1.6 ± 0.2 | 1.5 ± 0.4 | 1.4 ± 0.1 | 1.7 ± 0.1 | 1.3 ± 0.1 | 1.5 ± 0.1 |
| **TetraTetraAnh I** | 6.8 ± 0.9 | 5.2 ± 1.7 | 6.6 ± 0.4 | 6.5 ± 1.9 | 4.7 ± 2.0 | 5.6 ± 2.0 |
| **TetraTetraAnh II** | 3.2 ± 0.2 | 3.9 ± 1.6 | 3.4 ± 0.5 | 7.3 ± 0.4 | 4.7 ± 0.5 | 6.9 ± 0.3 |
| **TetraTetraTetraAnh I** | 1.7 ± 0.0 | 1.4 ± 0.4 | 1.9 ± 0.3 | 3.1 ± 0.3 | 2.2 ± 0.5 | 2.6 ± 0.4 |
| ***Summary***  **Monomers** | 54.7 ± 1.1 | 55.2 ± 2.3 | 54.6 ± 0.4 | 48.1 ± 1.7 | 65.1 ± 1.1 | 48.1 ± 0.5 |
| **Dimers** | 42.0 ± 0.9 | 41.9 ± 1.6 | 42.4 ± 0.1 | 47.0 ± 1.3 | 31.4 ± 0.8 | 47.8 ± 0.1 |
| **Trimers** | 3.3 ± 0.2 | 2.9 ± 0.7 | 3.1 ± 0.4 | 4.9 ± 0.4 | 3.5 ± 0.2 | 4.1 ± 0.6 |
| **Dipeptide (total)** | 0.0 ± 0.0 | 1.7 ± 0.4 | 0.0 ± 0.0 | 0.0 ± 0.0 | 24.4 ± 0.0 | 0.0 ± 0.0 |
| **Tripeptide (total)** | 3.0 ± 0.8 | 7.6 ± 1.2 | 2.5 ± 0.6 | 6.6 ± 0.8 | 13.5 ± 0.7 | 10.1 ± 0.4 |
| **Tetrapeptide (total)** | 93.7 ± 2.5 | 88.2 ± 1.2 | 93.9 ± 0.3 | 92.4 ± 5.9 | 59.1 ± 3.9 | 88.6 ± 2.7 |
| **Pentapeptide (total)** | 3.3 ± 0.5 | 2.6 ± 0.7 | 3.7 ± 0.6 | 1.1 ± 0.2 | 3.1 ± 0.5 | 1.3 ± 0.3 |
| **Peptides in cross-linkage (%)** | 45.3 ± 1.1 | 44.8 ± 2.3 | 45.4 ± 0.4 | 51.9 ± 1.7 | 34.9 ± 1.1 | 51.9 ± 0.6 |
| **Average chain length (DS)** | 18.1 ± 1.3 | 19.9 ± 0.2 | 18.1 ± 0.4 | 12.6 ± 1.5 | 18.4 ± 3.1 | 14.0 ± 2.3 |

**S1 Table. Summary of the muropeptide composition of diverse *V. cholerae* strains.**

^1^ Values are means ± variation for two independent PG preparations. The relative peak areas were estimated as the percentage of all peaks.

^2^ Nomenclature of muropeptides as in [1].

^3^ The Di fraction of sample Δ*varA*-20 h was collected and confirmed by mass spectrometry. The measured neutral mass was 698.0079 amu, the theoretical mass of GlcNAc-MurNAc(red)-L-Ala-D-Glu (Di) is 698.2558 amu.

**Supporting Reference**

1. Glauner B. Separation and quantification of muropeptides with high-performance liquid chromatography. Anal Biochem. 1988;172(2):451-64.
