# Supplementary material for: The VarA-CsrA regulatory pathway influences cell shape in Vibrio cholerae
Source: PLoS Genet. 2022 Mar 28;18(3):e1010143. doi: 10.1371/journal.pgen.1010143 (PMC8989286; doi:10.1371/journal.pgen.1010143)
Supplement: S2 Table — (DOCX) [file pgen.1010143.s011.docx]

**S2 Table. Summary of the muropeptide composition of shape-restored mutants in the ∆*varA* background.**

| **Muropeptide^2^** | **Relative % of muropeptide^1^** | | | |
| --- | --- | --- | --- | --- |
|  | **WT** | **Δ*varA*** | **Δ*aspA* Δ*varA*** | **Δ*varA*-Tn A** |
| **Tri** | 2.1 | 2.6 | 3.3 ± 0.1 | 2.2 ± 0.6 |
| **TetraGly4** | 2.5 | 1.0 | 2.8 ± 0.8 | 1.8 ± 0.1 |
| **Tetra** | 35.2 | 22.5 | 26.9 ± 0.1 | 26.6 ± 0.0 |
| **Di** | 0.5 | 23.5 | 3.4 ± 0.1 | 3.3 ± 2.0 |
| **Penta** | 1.9 | 0.8 | 2.4 ± 0.5 | 4.2 ± 0.9 |
| ***unknown*** | 1.0 | 1.9 | 1.2 ± 0.0 | 1.1 ± 0.0 |
| **TetraTri(Dap)** | 3.5 | 4.7 | 3.3 ± 0.5 | 4.0 ± 1.7 |
| **TetraTri** | 3.4 | 2.3 | 2.5 ± 1.0 | 2.2 ± 0.6 |
| **TetraTetra** | 22.4 | 10.8 | 18.8 ± 0.5 | 18.2 ± 1.5 |
| **TetraPenta** | 1.0 | 0.4 | 1.2 ± 0.0 | 1.5 ± 0.4 |
| **TetraTetraTetra** | 1.2 | 0.7 | 1.3 ± 0.1 | 1.3 ± 0.1 |
| **TetraTetraAnh I** | 3.3 | 2.1 | 3.2 ± 0.1 | 3.1 ± 0.3 |
| **TetraTetraAnh II** | 5.2 | 3.5 | 5.0 ± 0.1 | 5.5 ± 0.1 |
| **TetraTetraTetraAnh I** | 2.1 | 1.5 | 2.2 ± 0.1 | 2.3 ± 0.3 |
| ***Summary***  **Monomers** | 50.5 | 66.8 | 51.6 ± 1.5 | 50.6 ± 1.6 |
| **Dimers** | 45.5 | 30.3 | 43.9 ± 1.4 | 44.6 ± 2.0 |
| **Trimers** | 3.9 | 2.8 | 4.6 ± 0.1 | 4.7 ± 0.4 |
| **Dipeptide (total)** | 0.5 | 23.5 | 3.4 ± 0.1 | 3.3 ± 2.0 |
| **Tripeptide (total)** | 10.6 | 11.4 | 13.2 ± 0.3 | 10.6 ± 2.7 |
| **Tetrapeptide (total)** | 86.0 | 57.2 | 78.6 ± 0.3 | 78.8 ± 0.7 |
| **Pentapeptide (total)** | 2.8 | 1.3 | 3.8 ± 0.6 | 6.4 ± 0.8 |
| **Peptides in cross-linkage (%)** | 49.5 | 33.2 | 48.4 ± 1.5 | 49.4 ± 1.6 |
| **Average chain length (DS)** | 17.1 | 23.9 | 16.0 ± 0.1 | 15.2 ± 0.3 |

^1^ Values are means ± variation for two independent PG preparations, except for the WT and Δ*varA* strains, which are derived from a single experiment, as these strains have been previously analyzed (see Table S1). The relative peak areas were estimated as the percentage of all peaks.

^2^ Nomenclature of muropeptides as in [1].

**Supporting Reference**

1. Glauner B. Separation and quantification of muropeptides with high-performance liquid chromatography. Anal Biochem. 1988;172(2):451-64.
