# Supplementary material for: The VarA-CsrA regulatory pathway influences cell shape in Vibrio cholerae
Source: PLoS Genet. 2022 Mar 28;18(3):e1010143. doi: 10.1371/journal.pgen.1010143 (PMC8989286; doi:10.1371/journal.pgen.1010143)
Supplement: S4 Table — (DOCX) [file pgen.1010143.s013.docx]

**S4 Table. Bacterial strains used in this study**

| **Strains** | **Genotype and description** | **Internal number** | **Reference** |
| --- | --- | --- | --- |
| ***Vibrio cholerae*** | | | |
| WT | Wild-type A1552 O1 El Tor Inaba; Rif^R^ | GC#1 | [1] |
| Δ*varA* | A1552 deleted for *varA* (*VC1213*) via TransFLP; Rif^R^ | GC#3812 | This study |
| Δ*varA*-Tn-GFP | A1552Δ*varA* containing mini-Tn7-GFP; Cm^R^, Rif^R^, Gent^R^ | GC#9580 | This study |
| Δ*varA*+*varA* | A1552Δ*varA* containing mini-Tn7-*varA*; Rif^R^, Gent^R^ | GC#9263 | This study |
| Δ*varS* | A1552 deleted for *varS* (*VC2453*) via TransFLP; Rif^R^ | GC#3814 | This study |
| Δ*varS*Δ*varA* | A1552Δ*varS* deleted for *varA* (*VC1213*) using suicide plasmid pGP704-Sac28-Δ*varA*; Rif^R^ | GC#9264 | This study |
| Δ*hapR* | A1552Δ*hapR* (*VC0583*) | GC#3 | [2] |
| Δ*hapR*Δ*varA* | A1552Δ*hapR* deleted for *varA* (*VC1213*) using suicide plasmid pGP704-Sac28-Δ*varA*; Rif^R^ | GC#9265 | This study |
| Δ*luxO* | A1552Δ*luxO* (*VC1021*); Rif^R^ | GC#20 | [2] |
| Δ*luxO*Δ*varA* | A1552Δ*luxO* deleted for *varA* (*VC1213*) via TransFLP; Rif^R^ | GC#9267 | This study |
| Δ*ampG* | A1552 deleted for *ampG* (*VC2300*) via TransFLP; Rif^R^ | GC#9275 | This study |
| Δ*ampG*Δ*varA* | A1552Δ*ampG* deleted for *varA* (*VC1213*) via TransFLP; Rif^R^ | GC#9277 | This study |
| Δ*aspA* | A1552 deleted for *aspA* (*VC2698*) via TransFLP; Rif^R^ | GC#9269 | This study |
| Δ*aspA*Δ*varA* | A1552Δ*aspA*::FRT deleted for *varA* (*VC1213*) via TransFLP; Rif^R^ | GC#9272 | This study |
| Δ*aspA*Δ*varA*+*aspA* | A1552Δ*aspA*Δ*varA* containing mini-Tn7-*aspA*; Rif^R^, Gent^R^ | GC#9273 | This study |
| *luxO*[G333S] | A1552 with site-directed point mutation in *luxO* (resulting in LuxO[G333S]) | GC#4492 | [3] |
| *luxO*[G333S]Δ*var*A | A1552-*luxO*[G333S] deleted for *varA* (*VC1213*) via TransFLP; Rif^R^ | GC#9284 | This study |
| C6706-original | Wild-type C6706; O1 El Tor Inaba; non-mutated *luxO*; Str^S^ | GC#4522 | Gift from J. Mekalanos; [3]; |
| C6706Δ*varA* | C6706 deleted for *varA* (*VC1213*) via TransFLP; Str^S^ | GC#9280 | This study |
| C6706  (here: C6706-mut) | C6706 lacZ+ strain with mutated *luxO*[G333S]; Str^R^ | GC#4524 | Gift from J. Mekalanos (before repair; see [4]) |
| C6706-mutΔ*varA* | C6706-mut deleted for *varA* (*VC1213*) via TransFLP; Str^R^ | GC#9282 | This study |
| E7946 | Wild-type E7946; O1 El Tor Ogawa; isolated in 1978, Bahrain; Str^R^ | GC#2600 | Gift from A. Camilli [5] |
| E7946Δ*varA* | E7946 deleted for *varA* (*VC1213*) via TransFLP; Str^S^ | GC#9278 | This study |
| E7946  (here: E7946-AC) | Wild-type E7946; O1 El Tor Ogawa; isolated in 1978, Bahrain; Str^R^ | GC#6824 | Gift from A. Camilli [5] |
| E7946Δ*varA*  (here: E7946-AC  Δ*varA*) | E7946 deleted for *varA* (*VC1213*); Str^R^ | GC#6825 | Gift from A. Camilli; [6] |
| SA5Y | Environmental *V. cholerae* isolate collected in Old Salinas River (CA, USA) in May 2004 | GC#353 | [7] |
| ***V. cholerae* transposon mutants** | | | |
| Δ*varA*-Tn A | A1552Δ*varA* carrying the *mariner*-based transposon inserted in *csrA;* clone A | GC#9285 | This study |
| Δ*varA*-Tn B | A1552Δ*varA* carrying the *mariner*-based transposon inserted in *csrA;* clone B | GC#9286 | This study |
| Δ*varA*-Tn C | A1552Δ*varA* carrying the *mariner*-based transposon inserted in the upstream region of *csrA;* clone C | GC#9287 | This study |
| Δ*varA*-Tn D | A1552Δ*varA* carrying the *mariner*-based transposon inserted in the upstream region of *csrA;* clone D | GC#9288 | This study |
| Δ*varA*-Tn E | A1552ΔvarA carrying the *mariner*-based transposon inserted in the upstream region of *csrA;* clone E | GC#9289 | This study |
| Δ*varA*-Tn F | A1552ΔvarA carrying the *mariner*-based transposon inserted in the upstream region of *csrA;* clone F | GC#9290 | This study |
| Δ*varA*-Tn G | A1552ΔvarA carrying the *mariner*-based transposon inserted in *VCA0113;* A to G mutation at position -21 of *csrA;* clone G | GC#9291 | This study |
| Δ*varA*-Tn H | A1552Δ*varA* carrying the *mariner*-based transposon inserted in *VC1311;* A197C mutation in *csrA* resulting in CsrA[Stop66S]; clone H | GC#9292 | This study |
| Δ*varA*-Tn I | A1552Δ*varA* carrying the *mariner*-based transposon inserted in *VC1751;* T124C mutation in *csrA* resulting in CsrA[V42G]; clone I | GC#9293 | This study |
| Δ*varA*-Tn J | A1552Δ*varA* carrying the *mariner*-based transposon inserted in the upstream region of *csrA;* clone J | GC#9294 | This study |
| ***Vibrio harveyi*** | | | |
| *Vibrio harveyi*  DSM-19623 | *Vibrio harveyi* WT strain from DSMZ | GC#3440 | DSMZ culture collection |
| ***Escherichia coli*** | | | |
| S17-1λpir | Tp^R^ Sm^R^ *recA thi pro hsdR-M+ RP4:2-Tc:Mu*: Km^R^ Tn7 (λ*pir*) | GC#648 | [8] |
| MG1655 | F- lambda- *ilvG- rfb-50 rph-1* (cured derivative of K-12) | GC#2904 | Laboratory collection |
| MFD*pir* | MG1655 RP4-2-Tc::[Mu1::*aac*(3)IV-Δ*aphA*- Δ*nic35*-ΔMu2::zeo] Δ*dapA*::(erm-pir) Δ*recA* | GC#4662 | [9] |

**Supporting References**

1. Yildiz FH, Schoolnik GK. Role of *rpoS* in stress survival and virulence of *Vibrio cholerae*. J Bacteriol. 1998;180(4):773-84.

2. Meibom KL, Blokesch M, Dolganov NA, Wu C-Y, Schoolnik GK. Chitin induces natural competence in *Vibrio cholerae*. Science. 2005;310(5755):1824-7.

3. Stutzmann S, Blokesch M. Comparison of chitin-induced natural transformation in pandemic *Vibrio cholerae* O1 El Tor strains. Environ Microbiol. 2020;22(10):4149-66.

4. Caro F, Caro JA, Place NM, Mekalanos JJ. Transcriptional Silencing by TsrA in the Evolution of Pathogenic *Vibrio cholerae* Biotypes. mBio. 2020;11:e02901-20(6).

5. Miller VL, DiRita VJ, Mekalanos JJ. Identification of *toxS*, a regulatory gene whose product enhances ToxR-mediated activation of the cholera toxin promoter. J Bacteriol. 1989;171(3):1288-93.

6. Kamp HD, Patimalla-Dipali B, Lazinski DW, Wallace-Gadsden F, Camilli A. Gene Fitness Landscapes of *Vibrio cholerae* at Important Stages of Its Life Cycle. PLoS Pathog. 2013;9(12):e1003800.

7. Keymer DP, Miller MC, Schoolnik GK, Boehm AB. Genomic and phenotypic diversity of coastal *Vibrio cholerae* strains is linked to environmental factors. Appl Environ Microbiol. 2007;73(11):3705-14.

8. Simon R, Priefer U, Pühler A. A broad host range mobilization system for *in vivo* genetic engineering: transposon mutagenesis in Gram negative bacteria. Nat Biotechnol. 1983;1:784-91.

9. Ferrières L, Hemery G, Nham T, Guerout AM, Mazel D, Beloin C, et al. Silent mischief: bacteriophage Mu insertions contaminate products of *Escherichia coli* random mutagenesis performed using suicidal transposon delivery plasmids mobilized by broad-host-range RP4 conjugative machinery. J Bacteriol. 2010;192(24):6418-27.
