# Supplementary material for: The VarA-CsrA regulatory pathway influences cell shape in Vibrio cholerae
Source: PLoS Genet. 2022 Mar 28;18(3):e1010143. doi: 10.1371/journal.pgen.1010143 (PMC8989286; doi:10.1371/journal.pgen.1010143)
Supplement: S5 Table — (DOCX) [file pgen.1010143.s014.docx]

**S5 Table: Plasmids used in this study.**

| **Plasmid** | **Genotype** | **Internal number** | **Reference** |
| --- | --- | --- | --- |
| pBR-FRT-Kan-FRT2 | pBR322 derivative containing improved FRT-*aph*-FRT cassette, used as template for TransFLP; Amp^R^, Kan^R^ | GC#3782 | [1] |
| pBR-flp | pBR322 derivative containing FLP+, λ cI857+, λ pR from pCP20 integrated into the *EcoRV* site of pBR322; used for FLP recombination; Amp^R^ | GC#1203 | [2] |
| pUX-BF13 | pUX-BF13-oriR6K, helper plasmid with Tn7 transposition function; Amp^R^ | GC#457 | [3] |
| pGP704-mTn7 | pGP704 with mini-Tn7; Amp^R^, Gent^R^ | GC#645 | [4] |
| pGP704-Sac28 | suicide vector, *ori R6K*, *sacB*; Amp^R^ | GC#649 | [5] |
| pSC189 | Plasmid for the delivery of *mariner*-based transposon; Amp^R^, Kan^R^ | GC#6089 | [6] |
| pGP704-Sac28Δ*varA* | pGP704-Sac28 with *varA* gene fragment (Δ*varA*::FRT ; derived from the *varA* deletion via TransFLP) and its flanking regions; Amp^R^ | GC#9261 | This study |
| pGP704-mTn7-*varA* | pGP704 with mini-Tn7 carrying *varA* and its 164bp upstream region (with potential promoter region); Amp^R^ | GC#9260 | This study |
| pGP704-mTn7-*aspA* | pGP704 with mini-Tn7 carrying *aspA* and its 247bp upstream region (with potential promoter region); Amp^R^ | GC#9262 | This study |
| pGP704-mTn7-GFP | pGP704 with mini-Tn7 carrying P_A1/04/03_-GFPmut3^*^; Cm^R^, Strep^R^, Amp^R^, Gent^R^ | GC#458 | [4] |

**Supporting References**

1. Metzger LC, Stutzmann S, Scrignari T, Van der Henst C, Matthey N, Blokesch M. Independent Regulation of Type VI Secretion in *Vibrio cholerae* by TfoX and TfoY. Cell Rep. 2016;15(5):951-8.

2. De Souza Silva O, Blokesch M. Genetic manipulation of *Vibrio cholerae* by combining natural transformation with FLP recombination. Plasmid. 2010;64(3):186-95.

3. Bao Y, Lies DP, Fu H, Roberts GP. An improved Tn*7*-based system for the single-copy insertion of cloned genes into chromosomes of Gram-negative bacteria. Gene. 1991;109(1):167-8.

4. Müller J, Miller MC, Nielsen AT, Schoolnik GK, Spormann AM. *vpsA*- and *luxO*-independent biofilms of *Vibrio cholerae*. FEMS Microbiol Lett. 2007;275(2):199-206.

5. Meibom KL, Li XB, Nielsen AT, Wu CY, Roseman S, Schoolnik GK. The *Vibrio cholerae* chitin utilization program. Proc Natl Acad Sci USA. 2004;101(8):2524-9.

6. Chiang SL, Rubin EJ. Construction of a mariner-based transposon for epitope-tagging and genomic targeting. Gene. 2002;296(1-2):179-85.
